# Effect of tirzepatide treatment on patient-reported outcomes among SURMOUNT-OSA participants with obstructive sleep apnea and obesity

**DOI:** 10.1016/j.sleep.2025.106719

**Published:** 2025-08-06

**Authors:** Chisom Kanu, Shraddha Shinde, Sujatro Chakladar, Ellen B. Dennehy, Terri E. Weaver, Jiat Ling Poon, Atul Malhotra

**Affiliations:** a Eli Lilly and Company, Indianapolis, IN, USA; b College of Nursing, University of Illinois Chicago, Chicago, IL, USA; c University of Pennsylvania School of Nursing, Philadelphia, PA, USA; d University of California San Diego, La Jolla, CA, USA

**Keywords:** Obstructive sleep apnea, Obesity, Patient-reported outcomes, Health-related quality of life, Functional status

## Abstract

**Aim:**

In the phase 3 SURMOUNT-OSA trials, tirzepatide treatment significantly reduced the apnea-hypopnea index (AHI) among people with moderate-to-severe obstructive sleep apnea (OSA) and obesity. We evaluated effects of tirzepatide treatment on sleep disturbance, sleep-related impairment, functioning, health-related quality of life (HRQoL), and OSA symptoms in SURMOUNT-OSA participants.

**Methods:**

SURMOUNT-OSA consisted of two randomized, placebo-controlled trials of tirzepatide (10 mg or 15 mg) or placebo for 52 weeks in participants with moderate-to-severe OSA and obesity. For participants using PAP (Study 2), PAP was withdrawn prior to assessments of polysomnography and patient-reported outcome measures (PROMs). Prespecified PROM endpoints were from baseline to Week 52. Changes in sleep-related impairment, sleep disturbance, excessive daytime sleepiness, functioning, and HRQoL were assessed using analysis of covariance. Categorical shifts in OSA symptom severity were described.

**Results:**

At Week 52, compared with placebo, tirzepatide-treated participants reported significantly improved Patient-Reported Outcomes Measurement Information System (PROMIS) Short-Form Sleep-related Impairment 8a scores, PROMIS Short-Form v1.0 Sleep Disturbance 8b scores, Functional Outcomes of Sleep Questionnaire Activity-Level scores, EQ-5D-5L scores, and most domains of the Short-Form 36 Health Survey, Version 2. Tirzepatide treatment was also associated with greater improvements in Patient Global Impression of Status and Patient Global Impression of Change symptom scales compared with placebo. Additionally, Study 1 participants reported significant changes in Epworth Sleepiness Scale scores.

**Conclusion:**

Results indicate that in addition to objective outcomes of improved AHI, hypoxic burden associated with OSA, and cardiovascular risk factors, people with OSA reported benefits in symptoms, functioning, and HRQoL following tirzepatide treatment.

**Clinicaltrials.gov number:**

NCT05412004.

## 1. Introduction

Obstructive sleep apnea (OSA) is a sleep-related breathing disorder characterized by repetitive upper airway obstruction resulting in reduced (hypopnea) or absent (apnea) airflow, associated with cortical arousal or reduced blood oxygen saturation that leads to unrefreshing sleep, fatigue, tiredness, or lack of energy [1]. Globally, approximately 1 billion people aged 30–69 years have OSA, and around 425 million people have moderate-to-severe disease [2]. In the United States (US), approximately 39 million adults are estimated to have OSA [3]. OSA is associated with multiple complications and is an independent risk factor for cardiovascular diseases [4–6]. Moreover, a considerably higher proportion of people with obesity report OSA (40 %–70 %) than the general population [7]. OSA is also associated with poor quality of life, including excessive daytime sleepiness, difficulty in physical functioning, and cognitive impairment due to inadequate sleep [8–10].

Up to 90 % of people with OSA remain undiagnosed, possibly due to limited access to specialists, lack of a definitive diagnosis, the generic nature of the symptoms, and poor awareness of the serious consequences of OSA among patients [11,12]. Additionally, perceived lack of options for treatment of OSA, or undesirability of treatment options, may contribute to delay or avoidance of diagnosis [13]. The standard of care for OSA includes behavioral interventions (weight reduction, exercise, and sleep position restriction), medical devices (positive airway pressure [PAP] therapy or mandibular repositioning devices), and surgical procedures (such as maxillomandibular advancement or hypoglossal nerve stimulation) [1,5,14–17]. Most of the existing approved pharmacotherapies address only symptoms such as excessive sleepiness, rather than the underlying pathophysiology of the disease.

Tirzepatide is a glucose-dependent insulinotropic polypeptide (GIP) receptor and glucagon-like peptide-1 (GLP-1) receptor agonist approved in several countries for the treatment of type 2 diabetes and weight management [18–20]. The efficacy and safety of tirzepatide for the treatment of moderate-to-severe OSA in people with obesity were recently assessed in two phase 3 studies conducted under a master protocol, collectively referred to as SURMOUNT-OSA (NCT05412004) [21]. In these studies, tirzepatide treatment was associated with significantly reduced apnea-hypopnea index (AHI), bodyweight, hypoxic burden, systolic blood pressure, and high-sensitivity C-reactive protein concentration compared with placebo [21]. Tirzepatide treatment was also associated with significantly alleviated sleep impairment and sleep disturbance [21]. Tirzepatide was recently approved by the US Food and Drug Administration for the treatment of moderate-to-severe OSA in adults with obesity [22].

Patient-reported outcome measures (PROMs) can provide important insights into patients’ perspectives on their health, well-being, and day-to-day functioning [23,24]. Given the considerable impact of OSA on patients’ function and quality of life, the current secondary analysis further evaluated the effect of tirzepatide treatment on sleep disturbance and sleep-related impairment, OSA symptoms, functioning, and health-related quality of life (HRQoL) in SURMOUNT-OSA participants with moderate-to-severe OSA and obesity.

## 2. Methods

### 2.1. Study design and procedures

The phase 3 SURMOUNT-OSA included two independent 52-week, multicenter, randomized, placebo-controlled trials (Study 1 and Study 2) that evaluated the efficacy and safety of the maximum tolerated dose (MTD; 10 mg or 15 mg) of once weekly (QW) tirzepatide among adults with moderate-to-severe OSA and obesity [21,25]. The trials were conducted between June 21, 2022 and March 29, 2024. Study 1 included participants who were unable or unwilling to use PAP therapy, and Study 2 included those who had been using PAP therapy for at least 3 months at the time of screening and planned to continue its use during the trial.

Participants in both trials were randomly assigned (1:1) to receive either tirzepatide or placebo subcutaneously QW for 52 weeks in addition to lifestyle intervention (including behavioral counseling, a calorie-deficit diet, and physical activity). Tirzepatide dose was gradually increased every 4 weeks to reach the maximum tolerated dose of 10 or 15 mg. Participants unable to tolerate the MTD were discontinued from treatment. Participants in Study 2 were required to suspend PAP therapy for 7 days before polysomnography (PSG) and PROM assessments at baseline, Week 20, and Week 52 to minimize the confounding effect of PAP therapy on PROM assessments [26].

The trials were conducted in accordance with the Good Clinical Practice guidelines of the International Council for Harmonisation and the principles of the Declaration of Helsinki. An institutional review board at each site approved study protocols and informed consent forms. Before any study procedures were performed, written informed consent was obtained from each participant at the time of study entry.

### 2.2. Study population

The trials included adults with a diagnosis of moderate-to-severe OSA (AHI ≥15 events per hour) and obesity (body mass index [BMI] ≥30 kg/m^2^). The full list of inclusion and exclusion criteria has been published previously [21].

### 2.3. PROMs and study outcomes

Prespecified secondary and exploratory PROMs in SURMOUNT-OSA were measured at several timepoints in the study, concurrent with PSG assessment. For Study 2 participants, PROMs measured at baseline, Week 20, and Week 52 were preceded by PAP withdrawal procedures to minimize the confounding effect of PAP use. Thus, only changes from baseline to Week 20 (exploratory) and Week 52 (prespecified) were compared in Study 1 and Study 2. Concepts measured are listed below, with details presented in the Supplementary Materials.

#### (1) Changes in sleep-related impairment and sleep disturbance:

- The Patient-Reported Outcomes Measurement Information System (PROMIS) Short-Form Sleep-related Impairment 8a (PROMIS-SRI) assesses self-reported perceptions of alertness, sleepiness, and tiredness during usual waking hours in the past 7 days. Higher scores indicate more sleep-related impairment.
- The PROMIS Short-Form v1.0 Sleep Disturbance 8b (PROMIS-SD) consists of 8 items and assesses self-reported perceptions of sleep quality, sleep depth, and restoration associated with sleep in the past 7 days. Higher scores indicate more sleep disturbance.

#### (2) Change in excessive daytime sleepiness:

The Epworth Sleepiness Scale (ESS) assesses participants’ usual chances of dozing in different daytime situations in recent times. Higher scores indicate greater daytime sleepiness.

#### (3) Change in functional status:

The Functional Outcomes of Sleep Questionnaire (FOSQ) evaluates the effect of disorders associated with excessive daytime sleepiness on daily functioning in adults in various domains. The FOSQ 30-item Total score and the Total score for the FOSQ 10-item short-form (FOSQ-10) were calculated. Higher scores indicate better functional status.

#### (4) Change in HRQoL:

- The Short-Form 36 Health Survey, Version 2 (SF-36v2) acute form evaluates 8 physical and psychosocial domains, from which information is aggregated into Physical Component Summary and Mental Component Summary scores. The Physical Functioning domain assesses limitations due to health “now,” while the remaining domains assess functioning “in the past week.” Higher scores indicate better levels of function or better health.
- EQ-5D-5 Level (EQ-5D-5L) provides a single index value for health status in 5 dimensions as well as a single health state index value. The Health State Index ranges from less than 0 (where 0 is a health state equivalent to death) to 1 (perfect health). The EQ Visual Analog Scale that records the respondent’s self-rated health status from 0 (the worst imaginable health) to 100 (the best imaginable health) is also reported.

#### (5) Categorical shifts in OSA symptom severity:

- Patient Global Impression of Status (PGIS)-OSA scales: PGIS-OSA Fatigue, PGIS-OSA Sleepiness, and PGIS-OSA Snoring “over the past 7 days.” In addition, the global impression for sleep quality (PGIS Sleep Quality) was assessed using Item 8 of the PROMIS-SD.
- Patient Global Impression of Change (PGIC)-OSA scales: PGIC-OSA Fatigue, PGIC-OSA Sleepiness, PGIC-OSA Sleep Quality, and PGIC-OSA Snoring for the time period since the participant started the study medication.

### 2.4. Statistical analyses

Statistical analyses were performed in the modified intention-to-treat population (all participants who received at least one dose of tirzepatide or placebo, regardless of whether they discontinued the treatment or placebo for any reason) using the full analysis set for the treatment-regimen estimand, which represented the average treatment effect of tirzepatide versus placebo.

Changes in sleep-related impairment, sleep disturbance, excessive daytime sleepiness, functional status, and HRQoL were assessed at Weeks 20 and 52 using analysis of covariance (ANCOVA) with multiple imputation of missing values. The ANCOVA included the treatment and stratification factors (geographic region, sex, AHI stratum) as fixed effects and baseline as a covariate. For the single-item PGIS-OSA and PGIC-OSA symptom scales, the proportions of participants with improvements from baseline were summarized and shift analyses from baseline to Weeks 20 and 52 were performed. Graphical representations of the categorical shift in OSA symptom severity from baseline to Weeks 20 and 52 are presented.

For continuous measures, summary statistics included sample size, mean, and standard deviation (SD) for the actual and the change from baseline measurements. Least-square means (LSM) and standard errors (SE) for the change from baseline measurements were derived from the analysis models. Treatment comparisons were presented as the LSM change difference and 95 % confidence intervals (CI) with the p-values. Summary statistics included sample size, frequency, and percentages for categorical measures. Statistical comparisons between tirzepatide MTD and placebo groups were performed at a two-sided alpha level of 0.05.

## 3. Results

### 3.1. Participant characteristics

Demographics and key characteristics of SURMOUNT-OSA participants have been reported previously [21]. Study 1 included 234 randomized participants (mean age: 47.9 years; males: 67.1 %; mean BMI: 39.1 kg/m^2^; mean AHI: 51.5 events per hour), and Study 2 included 235 randomized participants (mean age: 51.7 years; males: 72.3 %; mean BMI: 38.7 kg/m^2^; mean AHI: 49.5 events per hour) [21].

At baseline, in both studies, there were no significant differences between the tirzepatide and placebo groups for all PROMs, except ESS scores in Study 2 (LSM difference for tirzepatide vs. placebo: 1.51; 95 % CI: 0.27, 2.76; p = 0.018) and SF-36v2 Social Domain scores in Study 1 (LSM difference for tirzepatide vs. placebo: 2.51; 95 % CI: 0.09, 4.94; p = 0.042).

### 3.2. Changes in sleep-related impairment and sleep disturbance

At Week 20, participants in the tirzepatide groups of both studies had greater reductions in PROMIS-SD (LSM difference [95 % CI] versus placebo: Study 1: 1.0 [−2.8,0.8]; Study 2: 2.8 [−4.9, −0.6]) and PROMIS-SRI scores (Study 1: 1.0 [−3.3, 1.2]; Study 2: 3.1 [−5.6, −0.5]). However, these changes were significant compared with placebo only in Study 2 (p < 0.05; Supplementary Fig. 2A).

**Fig. 1.**
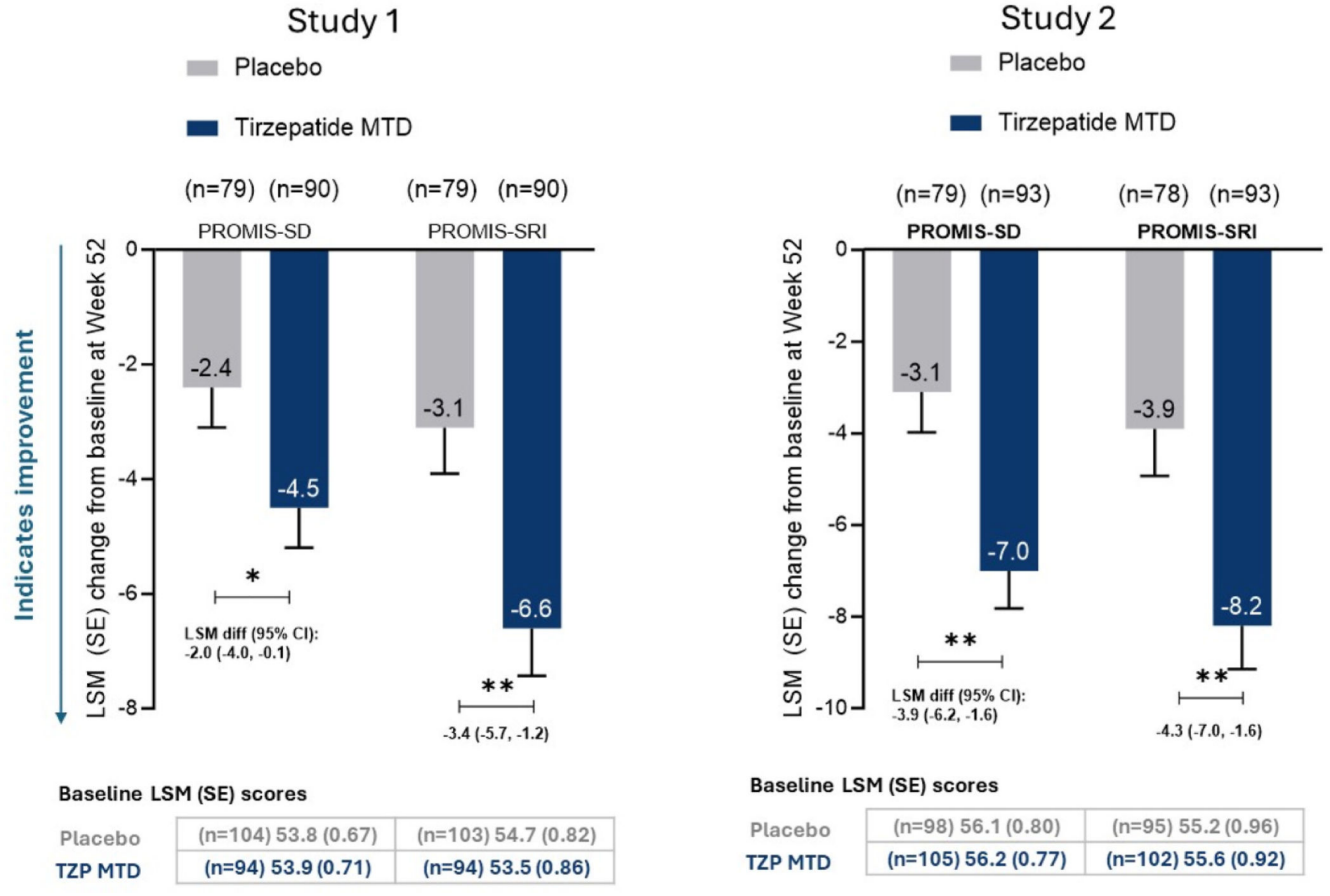
Change in patient-reported sleep disturbance and sleep-related impairment assessed by change in PROMIS-SD and PROMIS-SRI scores from baseline to Week 52 Abbreviations: CI, confidence interval; diff, difference; LSM, least squares mean; MTD, maximum tolerated dose, n, total number of patients with non-missing data at that particular timepoint; PROMIS-SRI, Patient-Reported Outcomes Measurement Information System Short-Form Sleep-related Impairment 8a; PROMI Data presented are least-square means derived using analysis of covariance with multiple imputation of missing values. The analysis included the full analysis set for the treatment-regimen estimand. **p-value <0.01, *p-value <0.05 versus placebo.

At Week 52, in both studies, participants in the tirzepatide groups reported significant reductions in PROMIS-SD and PROMIS-SRI scores versus placebo (Fig. 1). In Study 1, the LSM difference (95 % CI) for tirzepatide versus placebo was −2.0 (−4.0, −0.1) for PROMIS-SD and −3.4 (−5.7, −1.2) for PROMIS-SRI (p < 0.05 for both). In Study 2, the LSM difference (95 % CI) for tirzepatide versus placebo was −3.9 (−6.2, −1.6) for PROMIS-SD and −4.3 (−7.0, 1.6) for PROMIS-SRI (p < 0.05 for both).

### 3.3. Changes in other PROMs

**Fig. 2.**
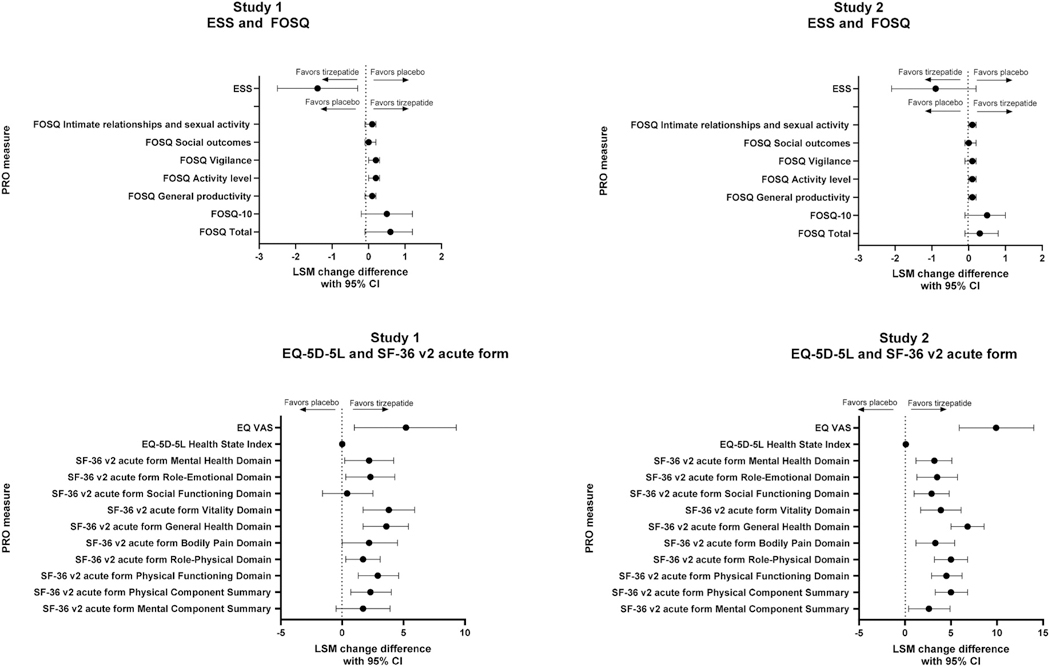
Forest plot of the treatment differences from baseline to Week 52 for the ESS, FOSQ, EQ-5D-5L, and SF-36 v2 acute form PROMs Abbreviations: CI, confidence interval; diff, difference; EQ-5D-5L, EQ-5D-5 Level; EQ-VAS, EQ -Visual Analog Scale; ESS, Epworth Sleepiness Scale; FOSQ, Functional Outcomes of Sleep Questionnaire; LSM, least squares mean; MTD, maximum tolerated dose; PROM, patient-reported outcome measures; SF-36 v2, Short-Form-36 Health Survey, Version 2; TZP, tirzepatide. Data presented are LSM change differences (95 % CI) for TZP MTD versus placebo derived using analysis of covariance with multiple imputation of missing values. The analysis included the full analysis set for the treatment-regimen estimand. Please refer to the Supplementary figures for the number of participants included in each PROM analysis.

The overall effects of tirzepatide treatment from baseline to Week 52 across the full set of PROMs are summarized in Fig. 2. The results of analyses of changes from baseline in PROMs assessing excessive daytime sleepiness, functional status, and HRQoL were directionally consistent, with tirzepatide treated patients showing greater improvement than those treated with placebo, often beginning before MTD was achieved (Supplementary Fig. 1A–D).

#### 3.3.1. Changes in excessive daytime sleepiness

At Week 20, in both studies, there were no significant differences between groups in the change from baseline in ESS scores (LSM difference [95 % CI] for tirzepatide versus placebo: Study 1: 0.6 [−1.7, 0.4]; Study 2: 1.0 [−2.1, 0.1]; p > 0.05; Supplementary Fig. 2B). At Week 52, in Study 1, participants in the tirzepatide group reported significant reductions in ESS scores versus placebo (LSM difference: 1.4; 95 % CI: 2.5, −0.3; p < 0.05; Fig. 2 and Supplementary Fig. 1A). In Study 2, there were no significant differences between groups in the change from baseline in ESS scores (LSM difference: 0.9; 95 % CI: 2.1, 0.2; p > 0.05).

#### 3.3.2. Changes in functional status

At Week 20, in Study 1, there were significant improvements in tirzepatide-treated participants compared to placebo in the Vigilance domain score (LSM difference [95 % CI]: 0.1 [0.0, 0.3]) and FOSQ-10 score (0.7 [0.1, 1.4]; p < 0.05 for both; Supplementary Fig. 2C). In Study 2, the tirzepatide group had significant improvements compared to placebo in General Productivity domain scores (LSM difference [95 % CI]: 0.1 [0.0, 0.2]), Activity level domain scores (0.1 [0.0, 0.2]), and FOSQ-10 scores (0.7 [0.1, 1.3]; p < 0.05 for all).

At Week 52, in Study 1 and Study 2, compared to placebo, there were no significant differences in FOSQ Total or FOSQ-10 scores. However, for both studies there were significant improvements in the FOSQ Activity-Level domain scores in tirzepatide-treated participants compared with placebo (LSM difference [95 % CI]: Study 1: 0.2 [0.0, 0.3]; Study 2: 0.1 [0.0, 0.2]; p < 0.05 for both; Fig. 2 and Supplementary Fig. 1B). Additionally, there were significant improvements in the FOSQ Vigilance domain score (Study 1, LSM difference [95 % CI]: 0.2 [0.0, 0.3]) and Intimate Relationships/Sexual Activity domain score (Study 2, 0.1 [−0.0, 0.2]) in the tirzepatide group compared with placebo (p < 0.05 for both). In both studies, there were no significant differences between the treatment groups in FOSQ Total scores, other domain scores, or FOSQ-10 scores.

#### 3.3.3. Changes in HRQoL

##### SF-36v2 acute form:

At Week 20, in Study 1, participants in the tirzepatide group reported statistically significant improvements versus placebo in the Physical Functioning (LSM difference [95 % CI]: 2.1 [0.5, 3.7]; p < 0.05), General Health (3.6 [1.5, 5.7]; p < 0.01), Social Functioning (2.0 [0.0,4.0]; p < 0.05) and Physical Component Summary scores (2.2 [0.5,3.9]; p < 0.05; Supplementary Fig. 2D). In Study 2, participants in the tirzepatide group reported statistically significant improvements versus placebo in all but the Mental Component Summary scores.

At Week 52, in Study 1, participants in the tirzepatide group reported statistically significant improvements in all but the Social Functioning domain and Mental Component Summary scores compared with placebo (Fig. 2 and Supplementary Fig. 1C). In Study 2, participants in the tirzepatide group reported statistically significant improvements in all domain and component summary scores at Week 52 versus placebo.

##### EQ-5D-5L:

At Week 52, compared with placebo, participants in the tirzepatide group reported significant improvements in EQ-5D-5L Health State Index (Study 1: LSM difference: 0.04; 95 % CI: 0.00, 0.08; Study 2: LSM difference: 0.05, 95 % CI: 0.01, 0.09; p < 0.05 for both; Fig. 2 and Supplementary Fig. 1D) and EQ-5D-5L VAS scores (Study 1: LSM difference: 5.2; 95 % CI: 1.0, 9.3; Study 2: LSM difference: 9.9, 95 % CI: 5.9, 14.0; p < 0.05 for both).

### 3.4. Changes in OSA symptoms

#### PGIS-OSA:

**Fig. 3.**
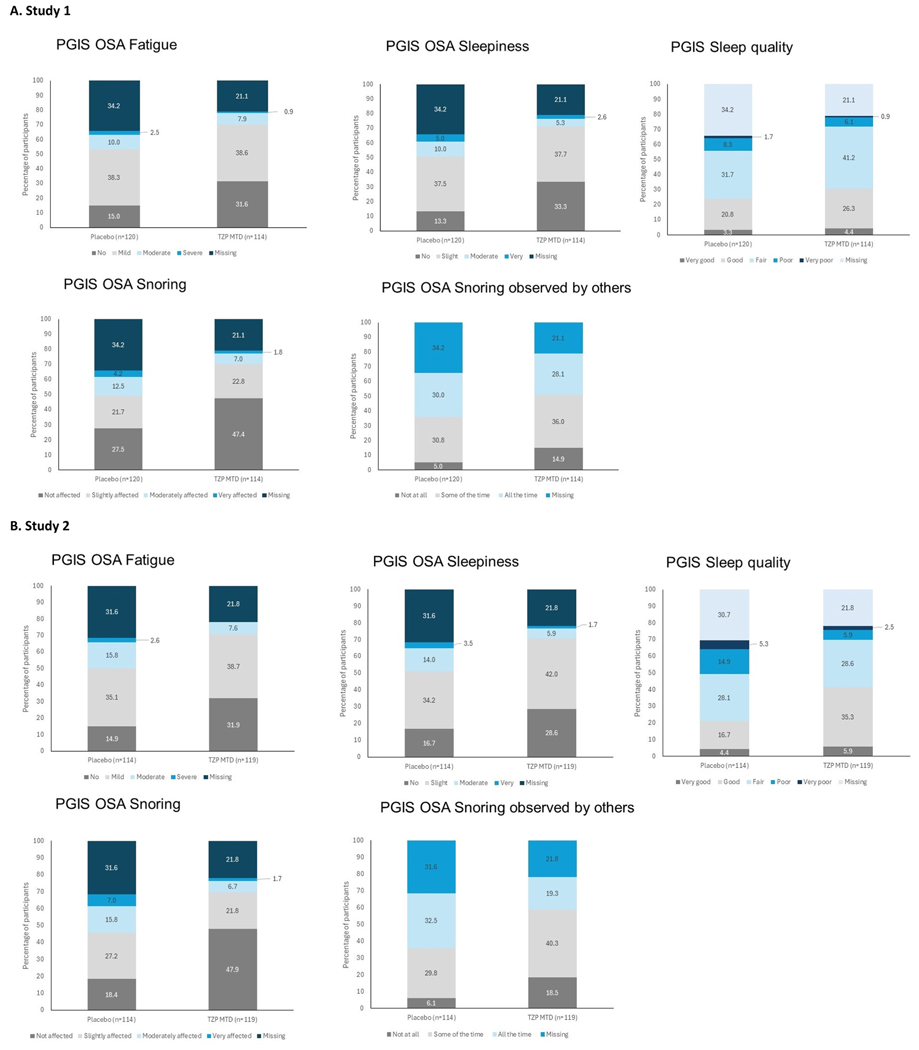
Shift plots of PGIS scales at Week 52 Abbreviations: MTD, maximum tolerated dose; n, number of participants in the population with baseline and postbaseline value; PGIS-OSA, Patient Global Impression of Status – Obstructive Sleep Apnea; TZP, tirzepatide.

In both studies, for each of the PGIS-OSA symptom scales related to sleepiness, fatigue, snoring, and sleep quality, participants in the tirzepatide group had greater shifts to an improved category than the placebo participants at Weeks 20 and 52 (Fig. 3, Supplementary Fig. 2E). More tirzepatide-treated participants reported less sleepiness during waking hours, fatigue, and snoring and had better sleep quality at Weeks 20 and 52 compared with the placebo group.

#### PGIC-OSA:

**Fig. 4.**
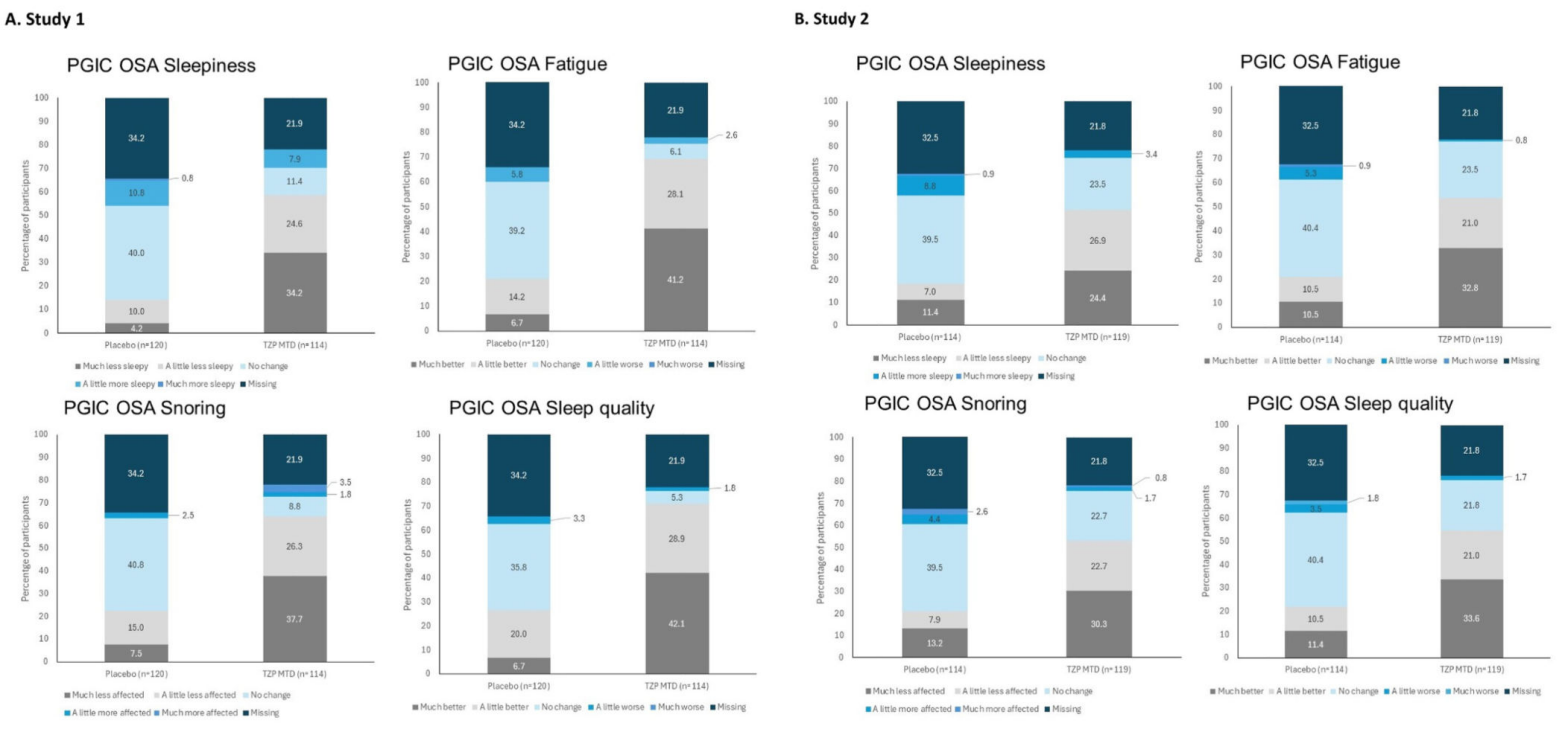
Shift plots of PGIC scales at Week 52 Abbreviations: MTD, maximum tolerated dose; n, number of participants in the population with baseline and postbaseline value; PGIC-OSA, Patient Global Impression of Change – Obstructive Sleep Apnea; TZP, tirzepatide.

Based on responses for the PGIC-OSA symptom scales related to sleepiness, fatigue, snoring, and sleep quality in both Study 1 and Study 2 at Weeks 20 and 52, more participants in the tirzepatide group reported that the change from baseline in their overall level of sleepiness and fatigue was better, that their sleep was less affected by snoring, and that they felt less sleepy during waking hours compared with the placebo group (Fig. 4, Supplementary Fig. 2F).

## 4. Discussion

In the SURMOUNT-OSA clinical trials, tirzepatide treatment was associated with improved quality of sleep, sleep-related functioning, and HRQoL among people with moderate-to-severe OSA and obesity. Significant benefits were observed following tirzepatide treatment compared with placebo in terms of sleep disturbance and sleep-related impairment (improved PROMIS-SD and PROMIS-SRI scores in both studies), excessive daytime sleepiness (significantly improved ESS scores in participants in Study 1), functional status domains (significant improvements in FOSQ Activity-Level and Vigilance domain scores in Study 1, Activity-Level and Intimate Relationships and Sexual Activity domain scores in Study 2), and HRQoL (most SF-36v2 domain scores in Study 1, all SF-36v2 domain and component summary scores in Study 2). Moreover, tirzepatide-treated participants reported improvements in symptom severity, with greater shifts to an improved category in PGIS-OSA and PGIC-OSA symptom scales compared with placebo in both studies. Some of these treatment benefits were seen quite early at Week 20 during dose escalation.

The Patient-Reported Outcomes Measurement Information System provides two measures derived from item banks related to sleep, the PROMIS-SRI and PROMIS-SD, which offer a means of assessing sleep-related impairment and sleep disturbance [27]. These two measures are not disease-specific, but rather provide general information regardless of underlying causes [27]. Donovan et al. previously reported that compared with ESS, the PROMIS-SD and PROMIS-SRI were more likely to detect an improvement in symptoms after PAP therapy initiation in people with type 2 diabetes who had OSA, suggesting that the PROMIS sleep measures may be sensitive to beneficial treatment effects in OSA [28]. We observed greater improvements in PROMIS-SD and PROMIS-SRI scores following tirzepatide treatment relative to placebo in both studies. The current analysis reports the first use of the PROMIS-SD and PROMIS-SRI measures in a large population of people with moderate-to severe OSA.

Excessive daytime sleepiness is one of the most common symptoms of OSA that impacts HRQoL and represents a safety risk for many patients. While the ESS has historically been used to assess changes in excessive daytime sleepiness, it has shown differential sensitivity across populations and lack of concordance between subjective reports of sleepiness with results of objective sleep latency tests [29–33]. Moreover, not all OSA patients report excessive daytime sleepiness [34,35]. Interestingly, only about half of the SURMOUNT-OSA study population endorsed excessive daytime sleepiness at baseline using the ESS, despite having significant disease as documented by AHI (baseline LSM scores: Study 1: 10.7 and 10.3; Study 2: 9.2 and 10.8 for placebo and tirzepatide MTD groups, respectively). Of note, participants were recruited from endocrinology and bariatric clinics, and not exclusively from sleep centers, and enrolment in SURMOUNT-OSA did not require a certain level of daytime sleepiness. Individuals who have baseline values indicating that they are not sleepy on the ESS are likely to remain non-sleepy following treatment and not report improvements on the measure. In the current analysis, tirzepatide-treated participants in Study 1 (without PAP therapy) showed significant improvements in ESS scores at Week 52 compared with placebo. In contrast, there was no significant difference in ESS score change at Week 52 between treatment groups in Study 2 (on PAP therapy). Despite PAP withdrawal prior to PROM assessments in Study 2, there may have been a residual impact of PAP use on the participants’ responses.

Current treatments for OSA have resulted in decreased functional impairment. The current analysis showed no differences in overall daily functioning as measured by the FOSQ Total score, and FOSQ-10 scores. However, there were significantly greater improvements observed with tirzepatide in FOSQ Activity-Level domain scores in both studies showing selective effect in accomplishing daily behaviors and keeping pace with peers. Greater alertness was demonstrated only in Study 2, which may be associated with a carry-over effect of PAP treatment. It is unclear why only those in Study 1 experienced greater intimacy and sexual activity. One possibility is a threshold effect in the PAP group at baseline for this domain limiting the level of improvement.

Conservative treatments such as sleep hygiene, positional therapy, physical exercise or weight reduction as well as surgical interventions have demonstrated improvements in HRQoL in people with OSA [28, 36–40]. Similarly, we observed significant improvements following tirzepatide treatment versus placebo in all but the Social Functioning and Mental Component Summary scores for the SF-36v2 domains indicating improved HRQoL. Additionally, the EQ-5D-5L and EQ-VAS scores in both studies at Week 52 also increased, indicating improved health status. Although these PROMs are not OSA-specific, the results may provide perspectives about the overall treatment-related changes in participants and enable cross-illness comparisons of effectiveness.

Participants receiving tirzepatide showed greater improvements in the PGIS-OSA symptom scales, less sleepiness during waking hours, less fatigue, snoring, and better sleep quality versus placebo. Similarly, more participants in the tirzepatide group reported improvements in these outcomes on the PGIC-OSA symptom scales compared with placebo. The benefits of tirzepatide treatment on the alleviation of symptom-related impairments have clinical relevance for disease management among people with OSA.

Despite the availability of several disease-specific and generic PROMs for use in sleep medicine, there has been limited use of PROMs in clinical research of patients with OSA [41]. PROMs can provide data from the patient’s perspective on the symptoms of OSA and its impact on their daily functioning, well-being, and HRQoL, which cannot be captured by objective sleep study assessments [42]. Assessing the impact of any treatment on the HRQoL of people with OSA is an important aspect of disease management as this can play a key role in treatment choice, treatment adherence, and patient satisfaction. The significant improvements across PROMs reported here demonstrate the potential of tirzepatide to improve how patients feel and function. The consistent trend of treatment-related improvements across multiple aspects of health suggests that these benefits were meaningful to patients. However, additional research is needed to determine thresholds for interpreting meaningful differences for these PROMs in patients with moderate-to-severe OSA and obesity, as there is scant literature on this topic. The varying approaches for determining and applying Minimum Important Difference (MID) thresholds in extant literature should be considered when comparing them to our study findings, as they can differ greatly based on the approach used and characteristics of the study sample [43,44]. Donovan et al. (2019) defined MID as half a standard deviation for the PROMIS-SRI, PROMIS-SD, and ESS at baseline, but used these thresholds (PROMIS-SD = 3.1; PROMIS-SRI = 4.9; ESS = 2.6) to define responders rather than interpret mean differences between groups [28]. Weaver et al. (2021) determined MID and clinically important response (CIR) thresholds for the FOSQ-10 score using anchor- and distribution-based analyses, then subsequently identified the MID (2.2) and CIR (1.8) thresholds with receiver operating characteristic analysis, concluding that these thresholds should be used to identify responders [45]. Despite the limited literature on meaningful change thresholds for the PROMs used in SURMOUNT-OSA, the study findings suggest that beyond the objective clinical metrics, patients with moderate-to-severe OSA perceived benefits from treatment, as shown by improvements across multiple PROM scores following treatment with tirzepatide [46].

### 4.1. Strengths and limitations

The current analysis has the following strengths. SURMOUNT-OSA was a large global study. Nearly one-third of the participants were women, a population typically underrepresented in OSA trials. Sleep-specific and generic PROMs were used to obtain a relatively broad representation of the burden of OSA on participants’ HRQoL. The trial design comprised two independent studies that included participants with and without current PAP therapy, thereby providing insights into the effect of tirzepatide treatment on OSA symptoms, functioning, and HRQoL in those populations that are predominant in clinical practice.

We acknowledge the following limitations. During sleep, people have limited self-awareness, thus, an accurate and complete description of sleep quality is challenging [27]. Additionally, it is difficult to specifically ascribe functional impairment to the quality or duration of sleep versus other potentially contributing causes. Given significant day-to-day variability in sleep-wake function, it is possible that assessment of sleep-related constructs would be best achieved using daily ratings, as opposed to evaluations at single points in time as utilized in SURMOUNT-OSA. Additionally, differing instructions across PROMs about the recall period (“in the last 7 days,” in recent times, etc.) may impact patient reports. Next, although OSA has a greater negative impact on the quality of life of women than men [9], a gender-based analysis was not in the scope of this analysis. The PROMs included in SURMOUNT-OSA may not have captured the full extent of patient-reported treatment benefits. Another limitation was studying individuals treated with PAP, which may account for the changes seen with tirzepatide treatment compared with placebo in ESS and FOSQ scores as seen in Study 1, versus the more limited differences observed in Study 2. Except for the PROMIS-SRI and PROMIS-SD, which were prespecified secondary endpoints, all other PROM endpoints were exploratory and may not have been adequately powered to detect differences between treatment groups.

Finally, there is limited data on clinically meaningful change thresholds for the PROMs that were used, and this may limit interpretation of our study findings in clinical practice, and available published thresholds should be interpreted within their given empirical context. The current paper is limited to results of prespecified statistical tests from baseline to Week 52 and exploratory tests from baseline to Week 20, evaluating the totality of the evidence showing convergence of beneficial effects across a number of outcomes meaningful to patients with OSA (Fig. 2). Further work to identify those outcomes that are most informative in studies of new treatments for OSA, and to determine clinically meaningful changes across groups, and within subjects, would be helpful to complement evaluation of PSG outcomes in studies of OSA.

Despite these limitations, we observed consistent benefits of tirzepatide treatment after 52 weeks of treatment, using a variety of PROMs that captured different constructs, including sleepiness, sleep-related impairment, sleep disturbance, and overall HRQoL, as visualized in Fig. 2. Quantitative results looking at change from baseline to Week 52 show statistically significant results in both studies for multiple outcomes, and the totality of evidence supports the benefit of tirzepatide treatment across a number of domains that are relevant for patients with OSA. However, thresholds for clinically relevant changes in the included PROMs have not yet been comprehensively examined in clinical practice. Therefore, the clinical importance of the observed benefits reported here needs to be further evaluated in future studies.

## 5. Conclusion

In the SURMOUNT-OSA clinical trials, tirzepatide was associated with directionally consistent improvement across most prespecified PROMs. Participants with moderate-to-severe OSA and obesity reported significant improvements in sleep disturbance, sleep-related impairment, functional status, activity levels, HRQoL, and symptom severity following 52 weeks of tirzepatide treatment compared with placebo. Some improvements were seen at Week 20 during dose titration. The beneficial impact of tirzepatide treatment on patient-reported outcomes and its reported improvements in AHI, bodyweight, and cardiovascular risk factors supports the benefits of tirzepatide treatment for adults with moderate-to-severe OSA and obesity [47,48].

## Supplementary Material

sup

## Data Availability

Eli Lilly and Company provides access to all individual participant data collected during the trial, after anonymization, except for pharmacokinetic or genetic data. Data are available to request 6 months after the indication studied has been approved in the USA and European Union and after primary publication acceptance, whichever is later. No expiration date of data requests is currently set once data have been made available. Access is provided after a proposal has been approved by an independent review committee identified for this purpose and after receipt of a signed data-sharing agreement. Data and documents, including the study protocol, statistical analysis plan, clinical study report and blank or annotated case report forms, will be provided in a secure data-sharing environment. For details on submitting a request, see the instructions provided at www.vivli.org.

## Appendix A. Supplementary data

Supplementary data to this article can be found online at https://doi.org/10.1016/j.sleep.2025.106719.

## Glossary

AHI: Apnea-Hypopnea Index
ANCOVA: Analysis of covariance
BMI: Body mass index
CI: Confidence interval
EQ-VAS: EuroQol Visual Analog Scale
ESS: Epworth Sleepiness Scale
FOSQ: Functional Outcomes of Sleep Questionnaire
GIP: Glucose-dependent insulinotropic polypeptide
GLP-1: Glucagon-like peptide-1
HRQoL: Health-Related Quality of Life
LSM: Least square means
MTD: Maximum tolerated dose
OSA: Obstructive Sleep Apnea
PAP: Positive Airway Pressure
PGIC-OSA: Patient Global Impression of Change for Obstructive Sleep Apnea
PGIS-OSA: Patient Global Impression of Severity for Obstructive Sleep Apnea
PROMIS-SD: Patient-Reported Outcomes Measurement Information System-Sleep Disturbance
PROMIS-SRI: Patient-Reported Outcomes Measurement Information System-Sleep Related Impairment
PROMs: Patient-Reported Outcome Measures
QW: Once weekly
SE: Standard error
SF-36v2: Short Form-36 Health Survey version 2
US: United States

## References

[1] Gottlieb DJ, Punjabi NM. Diagnosis and management of obstructive sleep apnea: a review. JAMA 2020;323(14):1389–400. 32286648 10.1001/jama.2020.3514

[2] Benjafield AV, Ayas NT, Eastwood PR, Estimation of the global prevalence and burden of obstructive sleep apnoea: a literature-based analysis. Lancet Respir Med 2019;7(8):687–98. 31300334 10.1016/S2213-2600(19)30198-5 PMC7007763

[3] Ling V Sleep apnea statistics and facts you should know. National Council on Aging. https://www.ncoa.org/adviser/sleep/sleep-apnea-statistics/. [Accessed 16 January 2025].

[4] Strenth C, Wani A, Alla R, Khan S, Schneider FD, Thakur B. Obstructive sleep apnea and its cardiac implications in the United States: an age-stratified analysis between young and older adults. J Am Heart Assoc 2024;13(12):e033810. 38842290 10.1161/JAHA.123.033810 PMC11255750

[5] Veasey SC, Rosen IM. Obstructive sleep apnea in adults. N Engl J Med 2019;380(15):1442–9. 30970189 10.1056/NEJMcp1816152

[6] Eshera YM, Gavrilova L, Hughes JW. Sleep is essential for cardiovascular health: an analytic review of the relationship between sleep and cardiovascular mortality. Am J Lifestyle Med 2024;18(3):340–50. 38737888 10.1177/15598276231211846 PMC11082862

[7] Pugliese G, Barrea L, Laudisio D, Sleep apnea, obesity, and disturbed glucose homeostasis: epidemiologic evidence, biologic insights, and therapeutic strategies. Curr Obes Rep 2020;9(1):30–8. 31970714 10.1007/s13679-020-00369-y

[8] Krishnan S, Chai-Coetzer CL, Grivell N, Comorbidities and quality of life in Australian men and women with diagnosed and undiagnosed high-risk obstructive sleep apnea. J Clin Sleep Med 2022;18(7):1757–67. 35332868 10.5664/jcsm.9972 PMC9243270

[9] Silva GE, Goodwin JL, Vana KD, Quan SF. Obstructive sleep apnea and quality of life: comparison of the SAQLI, FOSQ, and SF-36 questionnaires. Southwest J Pulm Crit Care 2016;13(3):137–49. 27738560 10.13175/swjpcc082-16 PMC5058363

[10] Dzierzewski JM, Soto P, Vahidi N, Nord R. Clinical characteristics of older adults seeking hypoglossal nerve stimulation for the treatment of obstructive sleep apnea. Ear Nose Throat J 2024;103(2):Np118–np123. 34464165 10.1177/01455613211042126 PMC9195674

[11] Finkel KJ, Searleman AC, Tymkew H, Prevalence of undiagnosed obstructive sleep apnea among adult surgical patients in an academic medical center. Sleep Med 2009;10(7):753–8. 19186102 10.1016/j.sleep.2008.08.007

[12] Pendharkar SR, Blades K, Kelly JE, Perspectives on primary care management of obstructive sleep apnea: a qualitative study of patients and health care providers. J Clin Sleep Med 2021;17(1):89–98. 32975193 10.5664/jcsm.8814 PMC7849647

[13] Ye L, Li W, Willis DG. Facilitators and barriers to getting obstructive sleep apnea diagnosed: perspectives from patients and their partners. J Clin Sleep Med 2022;18 (3):835–41. 34672944 10.5664/jcsm.9738 PMC8883110

[14] Randerath W, de Lange J, Hedner J, Current and novel treatment options for obstructive sleep apnoea. ERJ Open Res 2022;8(2). 10.1183/23120541.00126-2022 PMC9234427 35769417

[15] Randerath W, Verbraecken J, de Raaff CAL, European respiratory society guideline on non-CPAP therapies for obstructive sleep apnoea. Eur Respir Rev 2021;30(162). 10.1183/16000617.0200-2021 PMC9489103 34853097

[16] Randerath WJ, Verbraecken J, Andreas S, Non-CPAP therapies in obstructive sleep apnoea. Eur Respir J 2011;37(5):1000–28. 21406515 10.1183/09031936.00099710

[17] Verbraecken J, Dieltjens M, Op de Beeck S, Vroegop A, Braem M, Vanderveken O, Randerath W. Non-CPAP therapy for obstructive sleep apnoea. Breathe (Sheff) 2022;18(3):220164. 36340820 10.1183/20734735.0164-2022 PMC9584565

[18] Mounjaro^®^ (tirzepatide). Prescribing information. Lilly USA. https://pi.lilly.com/us/mounjaro-uspi.pdf?s=pi. [Accessed 9 August 2024].

[19] Zepbound^®^ (tirzepatide) injection. Prescribing information. Lilly USA. Accessed August 9, 2024. https://pi.lilly.com/us/zepbound-uspi.pdf.

[20] France NL, Syed YY. Tirzepatide: a review in type 2 diabetes. Drugs 2024;84(2):227–38. 38388874 10.1007/s40265-023-01992-4

[21] Malhotra A, Grunstein RR, Fietze I, Tirzepatide for the treatment of obstructive sleep apnea and obesity. N Engl J Med 2024. 10.1056/NEJMoa2404881 PMC11598664 38912654

[22] U.S. Food and Drug Administration. FDA approves first medication for obstructive sleep apnea. https://www.fda.gov/news-events/press-announcements/fda-approves-first-medication-obstructive-sleep-apnea. [Accessed 17 January 2025].

[23] Mercieca-Bebber R, King MT, Calvert MJ, Stockler MR, Friedlander M. The importance of patient-reported outcomes in clinical trials and strategies for future optimization. Patient Relat Outcome Meas 2018;9:353–67. 30464666 10.2147/PROM.S156279 PMC6219423

[24] Langendoen-Gort M, Groeneveld L, Prinsen CAC, Patient-reported outcome measures for assessing health-related quality of life in people with type 2 diabetes: a systematic review. Rev Endocr Metab Disord 2022;23(5):931–77. 35779199 10.1007/s11154-022-09734-9 PMC9515038

[25] Malhotra A, Bednarik J, Chakladar S, Tirzepatide for the treatment of obstructive sleep apnea: rationale, design, and sample baseline characteristics of the SURMOUNT -OSA phase 3 trial. Contemp Clin Trials 2024;141:107516. 38547961 10.1016/j.cct.2024.107516 PMC11168245

[26] Schwarz EI, Stradling JR, Kohler M. Physiological consequences of CPAP therapy withdrawal in patients with obstructive sleep apnoea-an opportunity for an efficient experimental model. J Thorac Dis 2018;10(Suppl 1):S24–s32. 29445525 10.21037/jtd.2017.12.142 PMC5803046

[27] Buysse DJ, Yu L, Moul DE, Development and validation of patient-reported outcome measures for sleep disturbance and sleep-related impairments. Sleep 2010;33(6):781–92. 20550019 10.1093/sleep/33.6.781 PMC2880437

[28] Donovan LM, Yu L, Bertisch SM, Buysse DJ, Rueschman M, Patel SR. Responsiveness of patient-reported outcomes to treatment among patients with type 2 diabetes mellitus and OSA. Chest 2020;157(3):665–72. 31785255 10.1016/j.chest.2019.11.011 PMC7609963

[29] Chervin RD, Aldrich MS, Pickett R, Guilleminault C. Comparison of the results of the epworth sleepiness scale and the multiple sleep latency test. J Psychosom Res 1997;42(2):145–55. 9076642 10.1016/s0022-3999(96)00239-5

[30] Miletin MS, Hanly PJ. Measurement properties of the Epworth sleepiness scale. Sleep Med 2003;4(3):195–9. 14592321 10.1016/s1389-9457(03)00031-5

[31] Murray BJ. A practical approach to excessive daytime sleepiness: a focused review. Cancer Res J 2016;2016:4215938. 10.1155/2016/4215938 PMC4904525 27445538

[32] Olson LG, Cole MF, Ambrogetti A. Correlations among Epworth sleepiness scale scores, multiple sleep latency tests and psychological symptoms. J Sleep Res 1998; 7(4):248–53. 9844851 10.1046/j.1365-2869.1998.00123.x

[33] Sangal RB, Mitler MM, Sangal JM. Subjective sleepiness ratings (Epworth sleepiness scale) do not reflect the same parameter of sleepiness as objective sleepiness (maintenance of wakefulness test) in patients with narcolepsy. Clin Neurophysiol 1999;110(12):2131–5. 10616118 10.1016/s1388-2457(99)00167-4

[34] Oksenberg A, Goizman V, Eitan E, Nasser K, Gadoth N, Leppänen T. How sleepy patients differ from non-sleepy patients in mild obstructive sleep apnea? J Sleep Res 2022;31(1):e13431. 34327744 10.1111/jsr.13431

[35] Zhang D, Luo J, Qiao Y, Xiao Y. Continuous positive airway pressure therapy in non-sleepy patients with obstructive sleep apnea: results of a meta-analysis. J Thorac Dis 2016;8(10):2738–47. 27867549 10.21037/jtd.2016.09.40 PMC5107495

[36] Costa IOM, Cunha MO, Bussi MT, Cassetari AJ, Zancanella E, Bagarollo MF. Impacts of conservative treatment on the clinical manifestations of obstructive sleep apnea-systematic review and meta-analysis. Sleep Breath 2024;28(4):1563–74. 38642201 10.1007/s11325-024-03034-z

[37] Diecidue RJ, LaNoue MD, Manning EL, Huntley CT, Harrington JD. Comparing treatment effectiveness and patient-reported outcome measures of four treatment options for obstructive sleep apnea. J Oral Maxillofac Surg 2024. 10.1016/j.joms.2024.07.015 39163993

[38] Friedman M, Schalch P, Joseph NJ. Palatal stiffening after failed uvulopalatopharyngoplasty with the pillar implant system. Laryngoscope 2006; 116(11):1956–61. 17075397 10.1097/01.mlg.0000242119.92179.b6

[39] Olszewska E, Vasilenok N, Polecka A, Stróżyński A, Olszewska N, Rogowski M, Fiedorczuk P. Long-term outcomes of pharyngoplasty for obstructive sleep apnea syndrome. Otolaryngol Pol 2022;76(3):18–25. 35796393 10.5604/01.3001.0015.7672

[40] Patil SP, Ayappa IA, Caples SM, Kimoff RJ, Patel SR, Harrod CG. Treatment of adult obstructive sleep apnea with positive airway pressure: an American academy of sleep medicine systematic review, meta-analysis, and GRADE assessment. J Clin Sleep Med 2019;15(2):301–34. 30736888 10.5664/jcsm.7638 PMC6374080

[41] Pevernagie D, Bauters FA, Hertegonne K. The role of patient-reported outcomes in sleep measurements. Sleep Med Clin 2021;16(4):595–606. 34711384 10.1016/j.jsmc.2021.07.001

[42] Bhat A, Singh A, Durr ML, Chang JL. Patient reported outcome measures used in surgical evaluation of obstructive sleep apnea: a systematic review. Laryngoscope 2024. 10.1002/lary.31603 38982930

[43] Health measures. Meaningful change. https://www.healthmeasures.net/score-and-interpret/interpret-scores/219-meaningful-change/260-meaningful-change. [Accessed 14 May 2025].

[44] Wright A, Hannon J, Hegedus EJ, Kavchak AE. Clinimetrics corner: a closer look at the minimal clinically important difference (MCID). J Man Manip Ther 2012;20(3):160–6. 23904756 10.1179/2042618612Y.0000000001 PMC3419574

[45] Weaver TE, Menno DM, Bron M, Crosby RD, Morris S, Mathias SD. Determination of thresholds for minimally important difference and clinically important response on the functional outcomes of sleep questionnaire short version in adults with narcolepsy or obstructive sleep apnea. Sleep Breath 2021;25(3):1707–15. 33394323 10.1007/s11325-020-02270-3 PMC8376693

[46] Fredericksen RJ, Yang FM, Gibbons LE, Development and content validation of measures assessing adherence barriers and behaviors for use in clinical care. Res Soc Adm Pharm 2019;15(9):1168–76. 10.1016/j.sapharm.2018.10.001 PMC7946330 30327183

[47] Lisik D, Pires GN, Zou D. Perspective: systematic review and meta-analysis in obstructive sleep apnea - what is lacking? Sleep Med 2023;111:54–61. 37717377 10.1016/j.sleep.2023.09.006

[48] Lisik D, Zou D. Breaking ground: from CPAP treatment to the first medicine for OSA patients with obesity. Curr Pulmonol Rep 2025;14(1):3.

