# Supplementary material for: Effect of tirzepatide treatment on patient-reported outcomes among SURMOUNT-OSA participants with obstructive sleep apnea and obesity
Source: Sleep Med. Author manuscript; Available in PMC 2025 Dec 22. (PMC12720398; doi:10.1016/j.sleep.2025.106719)
Supplement: sup [file NIHMS2103993-supplement-sup.pdf]

Supplementary data

Supplementary methods: PROMs

PROM	Description
Patient-Reported Outcomes Measurement Information System (PROMIS) Sleep Disturbance and PROMIS Sleep-related impairment	The PROMIS Short Form v1.0 Sleep Disturbance 8b assesses self-reported perceptions of sleep quality, sleep depth, and restoration associated with sleep, including perceived difficulties and concerns with getting to sleep or staying asleep, in addition to perceptions of the adequacy of and satisfaction with sleep. The PROMIS Short Form v1.0 Sleep Disturbance 8b consists of 8 items each rated on a 5-point scale ranging from "not at all" to "very much, " "never" to "always," or "very poor" to "very good." Items have a recall period of "in the past 7 days." Individual item scores are totaled to obtain a raw score, with higher scores indicating more sleep disturbance. Raw scores can be converted to a T-score, which is standardized with a mean of 50 and a standard deviation (SD) of 10.<br>The PROMIS Short Form v1.0 Sleep-related Impairment 8a assesses self-reported perceptions of alertness, sleepiness, and tiredness during usual waking hours, and the perceived functional impairments associated with sleep problems or impaired alertness. The PROMIS Short Form v1.0 Sleep-related Impairment 8a consists of 8 items each rated on a 5-point scale ranging from "not at all" to "very much." Items have a recall period of "in the past 7 days." Individual item scores are totalled to obtain a raw score, with higher scores indicating more sleep-related impairment. Raw scores can be converted to a T-score, which is standardized with a mean of 50 and a SD of 10.
Epworth Sleepiness Scale (ESS)	The ESS assesses improvements in excessive daytime sleepiness. This is an 8-item participant-completed measure that asks the participant to rate on a scale of 0 (would never doze) to 3 (high chance of dozing), their usual chances of dozing in 8 different daytime situations, with a recall period of "in recent times." The ESS total score is the sum of the 8-item scores and ranges from 0 to 24, with higher scores indicating greater daytime sleepiness.
Functional Outcomes of Sleep Questionnaire (FOSQ)	The FOSQ is a 30-item sleep-specific, participant-completed questionnaire used to assess the effect of disorders associated with excessive daytime sleepiness on daily functioning in adults. It assesses the following domains:<br>• General productivity (8 items)<br>• Activity level (9 items)<br>• Vigilance (7 items)<br>• Social outcomes (2 items)<br>• Intimate and sexual relationships (4 items)<br>The FOSQ items assess participants' current status with each item rated on a scale of 1 (extreme difficulty) to 4 (no difficulty), with an additional not applicable (0 = "I don't do this activity for other reasons") also available. Individual domain scores are calculated by taking the mean of answered, non-zero items within each domain and a total score can be calculated by first computing the mean score for each domain, then multiplying the mean of the domain scores by 5 (Weaver et al. 1997). The Total score for the FOSQ 10-item short-form (FOSQ-10) was also calculated.

PROM	Description
Short-Form 36 Version 2 Health Survey (SF-36v2), Acute Form, 1-week Recall Version	The SF-36v2 acute form, 1-week recall version is a 36-item generic, participant-completed measure designed to assess the following 8 domains.<br>• Physical functioning<br>• Role-physical<br>• Bodily pain<br>• General health<br>• Vitality<br>• Social functioning<br>• Role-emotional, and<br>• Mental health<br>The Physical Functioning domain assesses limitations due to health "now" while the remaining domains assess functioning "in the past week." Each domain is scored individually and information from these 8 domains is further aggregated into 2 health component summary scores: Physical Component Summary and Mental Component Summary. Items are answered on Likert scales of varying lengths (3-point, 5-point, or 6-point scales). Scoring of each domain and both summary scores are norm-based and presented in the form of T-scores, with a mean of 50 and SD of 10; higher scores indicate better levels of function and/or better health (Maruish 2011).
Patient Global Impression of Status – Obstructive Sleep Apnea (PGIS-OSA) Symptom Scales	This includes 3 patient global impression of status scales of OSA symptom severity.<br>*PGIS-OSA Fatigue:* This is a single-item, participant self-rated assessment of their overall level of fatigue due to OSA, "over the past 7 days." The item is rated on a 4-point scale ranging from "No fatigue" to "Severe fatigue."<br>*PGIS-OSA Sleepiness:* This is a single-item, participant self-rated assessment of their overall level of sleepiness due to OSA during waking hours, "over the past 7 days." The item is rated on a 4-point scale ranging from "Not at all sleepy" to "Very sleepy."<br>*PGIS-OSA Snoring:* The PGIS-OSA Snoring scale consists of two items. The first item is a participant self-rated assessment of their overall perception of the severity of their snoring due to OSA, "over the past 7 days," with respect to how much their snoring has affected their sleep. The item is rated on a 4-point scale ranging from "Not at all affected" to "Very affected." For the second item, participants will be asked on a 3-point scale ("Not at all" to "All the time") if they have ever been told by someone else that they snore in their sleep.<br>The global impression item for sleep quality (PGIS Sleep Quality) was also assessed using item 8 of the PROMIS-SD.

PROM	Description
EQ – 5D- 5 Level (EQ-5D-5L)	The EQ-5D-5L (EuroQol Research Foundation 2019) is a standardized 5-item self-administered instrument for use as a measure of health outcome. It provides a simple descriptive profile and a single index value for health status that can be used in the clinical and economic evaluation of health care as well as population health surveys. The EQ-5D-5L assesses 5 dimensions of health:<br>• mobility<br>• self-care<br>• usual activities<br>• pain/discomfort, and<br>• anxiety/depression.<br>The 5L version, scores each dimension at 5 levels:<br>• no problems<br>• slight problems<br>• moderate problems<br>• severe problems, and<br>• unable to perform/extreme problems.<br>A total of 3125 health states is possible. In addition to the health profile, a single health state index value can be derived based on a formula that attaches weights to each of the levels in each dimension. This index value ranges between less than 0 (where 0 is a health state equivalent to death; negative values are valued as worse than dead) to 1 (perfect health). In addition, the EQ Visual Analog Scale records the respondent's self-rated health status on a vertical graduated (0 to 100) visual analog scale. The participant rates his/her perceived health from 0 (the worst imaginable health) to 100 (the best imaginable health). In conjunction with the health state data, it provides a composite picture of the respondent's health status.
Patient Global Impression of Change – Obstructive Sleep Apnea (PGIC-OSA) Symptom Scales	Patient global impression of change scales assessed change in OSA symptom severity.<br>*PGIC-OSA Fatigue:* This is a single-item, participant self-rated assessment of the change in their overall level of fatigue due to OSA, "since you started taking the study medication." The item is rated on a 5-point scale ranging from "Much worse" to "Much better."<br>*PGIC-OSA Sleepiness:* This is a single-item, participant self-rated assessment of the change in their overall level of sleepiness due to OSA during waking hours, "since you started taking the study medication." The item is rated on a 5-point scale ranging from "Much more sleepy" to "Much less sleepy."<br>*PGIC-OSA Sleep Quality:* This is a single-item, participant self-rated assessment of the change in their overall sleep quality due to OSA, "since you started taking the study medication." The item is rated on a 5-point scale ranging from "Much worse" to "Much better."<br>*PGIC-OSA Snoring:* This is a single-item, participant self-rated assessment of the overall change in how their snoring has affected their sleep, "since you started taking the study medication." The item is rated on a 5-point scale ranging from "My sleep is much more affected" to "My sleep is much less affected."

Abbreviations: PROM, patient-reported outcome measure

Supplementary Figure 1: Change from baseline to Week 52 (prespecified)

A. Change in ESS scores

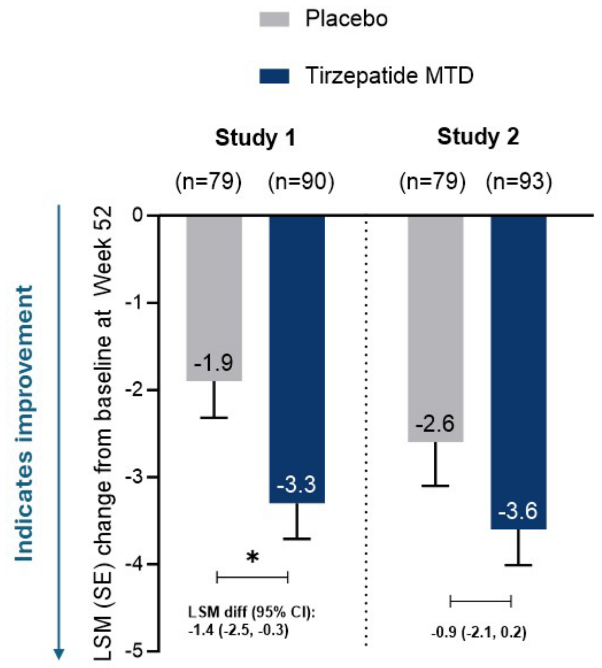

Baseline LSM (SE) scores

	Study 1	Study 2	
Placebo (n=107)	10.7 (0.50)	9.2 (0.45)	Placebo (n=102)
TZP MTD (n=96)	10.3 (0.53)	10.8 (0.44)[1]	TZP MTD (n=107)

[1] LSM difference for TZP MTD versus placebo = 1.51 (95% CI: 0.27, 2.76), p=0.018

Abbreviations: CI, confidence interval; diff, difference; ESS, Epworth Sleepiness Scale; LSM, least-square means; MTD, maximum tolerated dose; n, number of participants in the population with baseline and postbaseline value at the specified time point; N, number of participants in the analysis population; SE, standard error; TZP, tirzepatide.

Data presented are least-square means derived using analysis of covariance with multiple imputation of missing values. The analysis included the full analysis set for the treatment-regimen estimand.

*p-value <0.05 versus placebo

B. Change in FOSQ and FOSQ-10 scores

Study 1

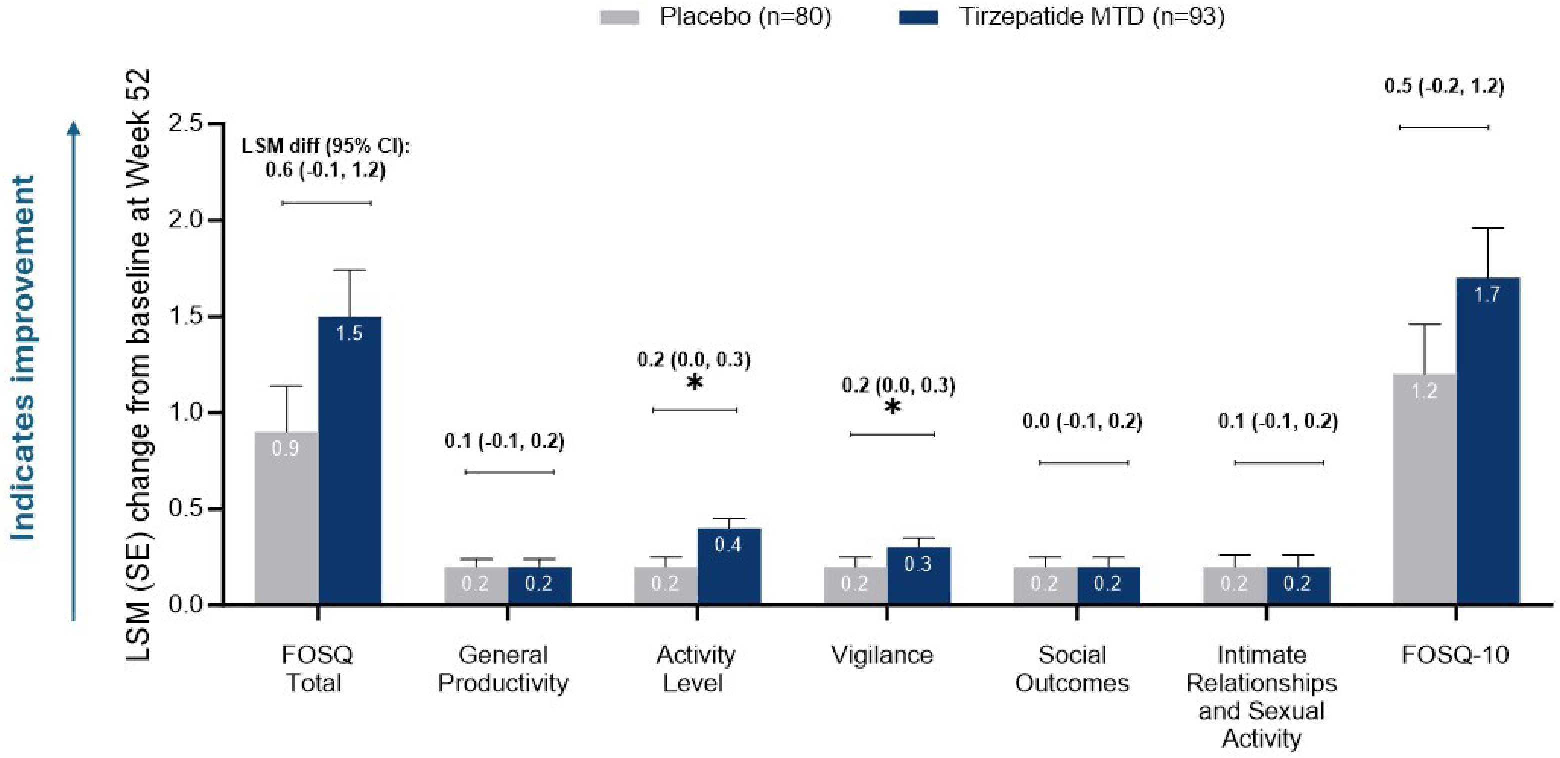

Baseline LSM (SE) scores

	FOSQ Total	General Productivity	Activity Level	Vigilance	Social Outcomes	Intimate Relationships and Sexual Activity	FOSQ-10
Placebo (n=118)	15.8 (0.27)	3.4 (0.05)	3.0 (0.06)	3.1 (0.06)	3.5 (0.07)	3.3 (0.07)	15.2 (0.28)
TZP MTD (n=110)	16.2 (0.28)	3.4 (0.05)	3.1 (0.06)	3.2 (0.07)	3.5 (0.07)	3.4 (0.08)	15.6 (0.29)

N values at baseline for Social outcomes: TZP MTD: n=108; Intimate Relationships and Sexual Activity: Placebo n=102, TZP MTD n=101
N values at Week 52 for Social outcomes: Placebo: n=78, TZP-MTD: n=92; Intimate Relationships and Sexual Activity: Placebo: n=68, TZP MTD: n=83

Study 2

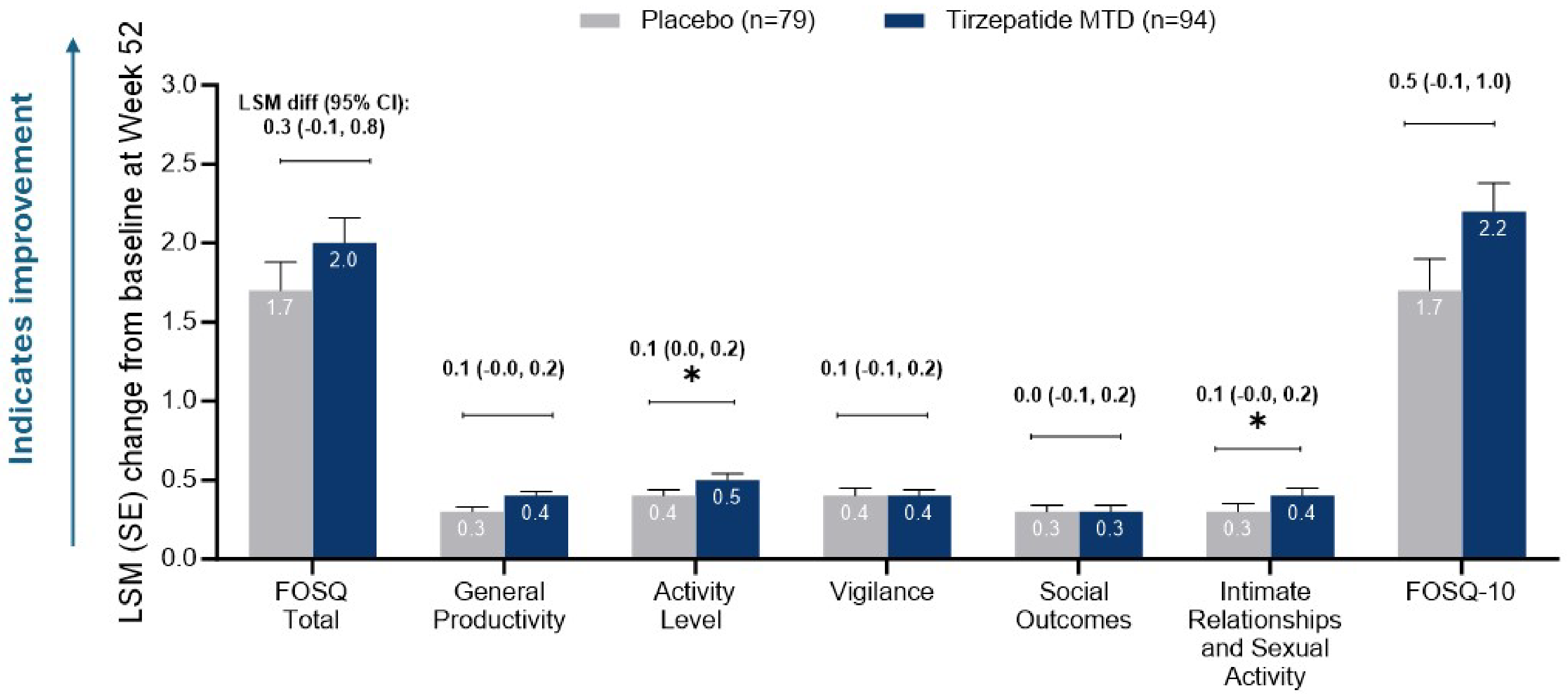

Baseline LSM (SE) scores

Placebo (n=112)	16.5 (0.27)	3.4 (0.05)	3.1 (0.06)	3.2 (0.06)	3.5 (0.07)	3.3 (0.08)	16.1 (0.29)
**TZP MTD (n=117)**	**16.3 (0.27)**	**3.4 (0.05)**	**3.0 (0.06)**	**3.1 (0.06)**	**3.5 (0.07)**	**3.3 (0.08)**	**15.8 (0.28)**

N values at baseline for Social outcomes: Placebo: n=109, TZP MTD: n=111; Intimate Relationships and Sexual Activity: Placebo: n=103, TZP MTD: n=102
N values at Week 52 for Social outcomes: Placebo: n=78, TZP-MTD: n=93; Intimate Relationships and Sexual Activity: Placebo: n=68, TZP MTD: n=80
Abbreviations: CI, confidence interval; diff, difference; FOSQ, Functional Outcomes of Sleep Questionnaire; LSM, least-square means; MTD, maximum tolerated dose; n, number of participants in the population with baseline and postbaseline value at the specified time point; N, number of participants in the analysis population; SE, standard error.

Data presented are least-square means derived using analysis of covariance with multiple imputation of missing values. The analysis included the full analysis set for the treatment-regimen estimand.
General productivity, Activity Level, Vigilance, Social Outcomes, and Intimate Relationships and Sexual Activity are all domains of FOSQ.
*p-value <0.05 versus placebo

C. Change in SF-36v2 scores

Study 1

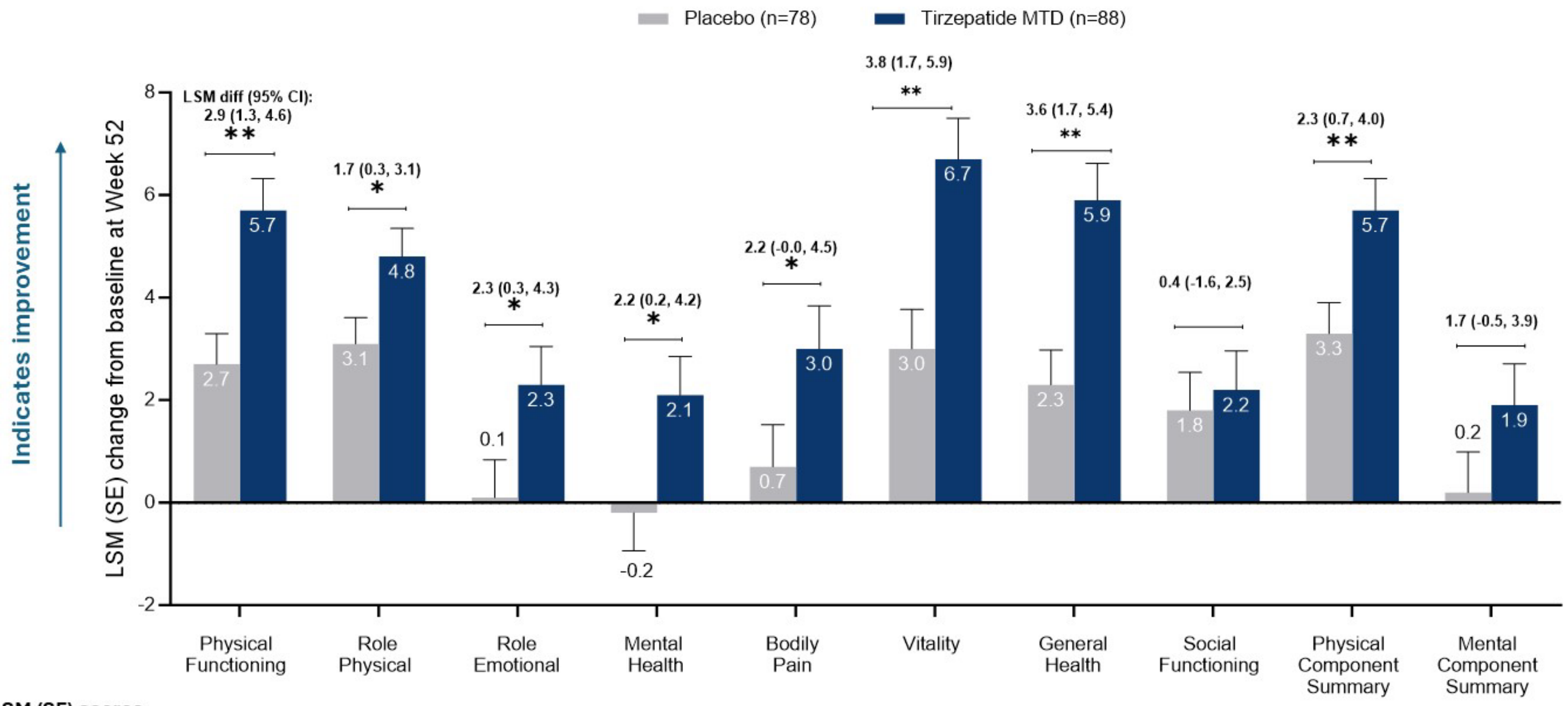

Baseline LSM (SE) scores

Placebo (n=99)	45.6 (0.83)	47.4 (0.75)	49.1 (0.88)	49.3 (0.86)	48.5 (0.95)	47.2 (0.94)	47.1 (0.88)	47.4 (0.85)	46.3 (0.79)	49.4 (0.92)
**TZP MTD (n=92)**	**47.2 (0.86)**	**49.2 (0.78)**	**49.5 (0.91)**	**49.1 (0.89)**	**50.0 (0.98)**	**48.0 (0.98)**	**46.4 (0.91)**	**49.9 (0.88)[1]**	**47.9 (0.81)**	**49.7 (0.95)**

[1] LSM difference for TZP MTD versus placebo = 2.5 (95% CI: 0.09, 4.94), p=0.042

Study 2

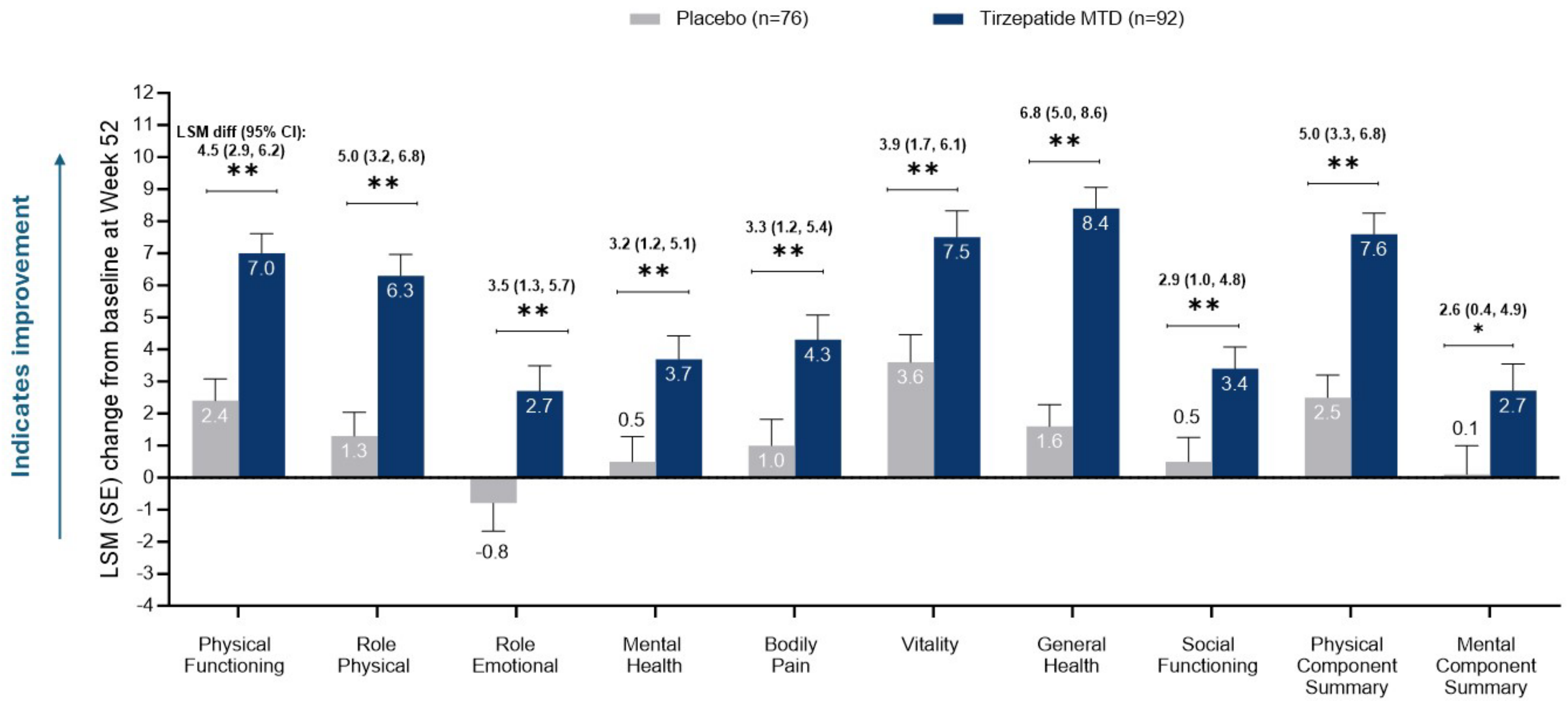

Baseline LSM (SE) scores

	Physical Functioning	Role Physical	Role Emotional	Mental Health	Bodily Pain	Vitality	General Health	Social Functioning	Physical Component Summary	Mental Component Summary
Placebo (n=87)	46.5 (0.93)	46.7 (0.89)	49.4 (0.88)	50.8 (0.83)	50.9 (1.00)	47.8 (1.02)	47.4 (0.99)	49.3 (0.88)	46.9 (0.92)	50.6 (0.90)
TZP MTD (n=87)	48.4 (0.93)	48.4 (0.89)	49.6 (0.88)	49.1 (0.83)	49.5 (1.00)	47.1 (1.02)	46.6 (0.99)	50.1 (0.88)	47.92 (0.92)	49.4 (0.90)

Abbreviations: CI, confidence interval; diff, difference; LSM, least-square means; MTD, maximum tolerated dose; n, number of participants in the population with baseline and postbaseline value at the specified time point; N, number of participants in the analysis population; SF-36 v2, Short-Form-36 Health Survey, Version 2; SE, standard error.

Data presented are least-square means derived using analysis of covariance with multiple imputation of missing values. The analysis included the full analysis set for the treatment-regimen estimand.

*p-value <0.05 versus placebo, **p-value <0.01 versus placebo

D. Change in EQ-5D-5L Health State Index and EQ-VAS scores

A. EQ-5D-5L Health State Index

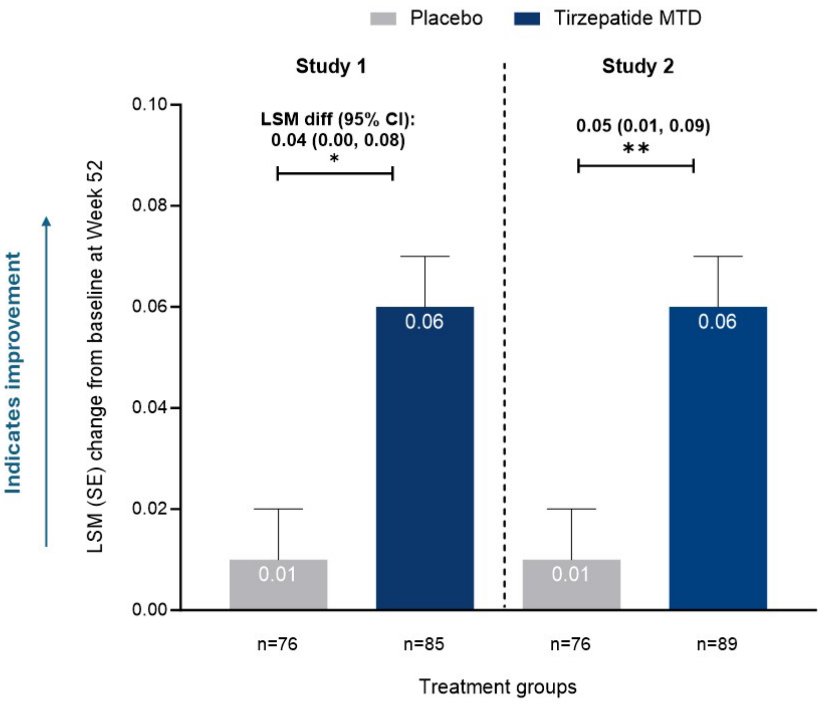

Baseline LSM (SE) scores

	Study 1	Study 2	
Placebo (n=96)	0.81 (0.02)	0.83 (0.01)	Placebo (n=90)
TZP MTD (n=88)	0.82 (0.02)	0.84 (0.01)	TZP MTD (n=96)

B. EQ-5D-5L VAS

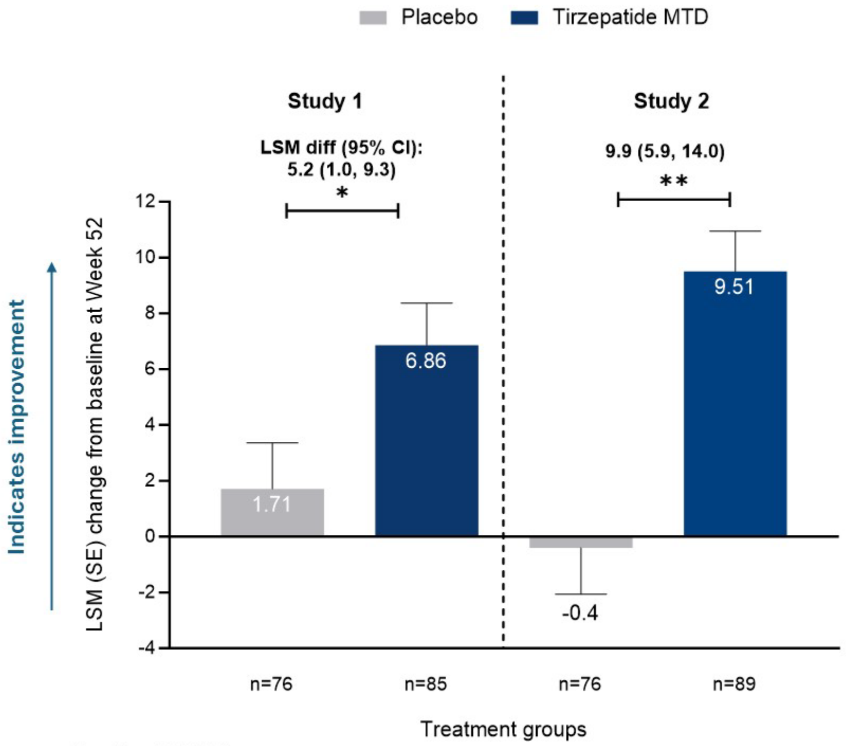

Baseline LSM (SE) scores

	Study 1	Study 2	
Placebo (n=96)	78.1 (1.56)	76.8 (1.75)	Placebo (n=90)
TZP MTD (n=88)	77.4 (1.62)	76.1 (1.69)	TZP MTD (n=96)

Abbreviations: CI, confidence interval; diff, difference; EQ-5D-5L, EQ-5D-5 Level; EQ-VAS, EQ -Visual Analog Scale; LSM, least-square means; MTD, maximum tolerated dose; n, number of participants in the population with baseline and postbaseline value; N, number of participants in the analysis population; SE, standard error.

Data presented are least-square means derived using analysis of covariance with multiple imputation of missing values. The analysis included the full analysis set for the treatment-regimen estimand.

*p-value <0.05 versus placebo, **p-value <0.01 versus placebo.

Supplementary Figure 2: Change from baseline to Week 20 (exploratory)

A. Change in PROMIS-SD and PROMIS-SRI scores

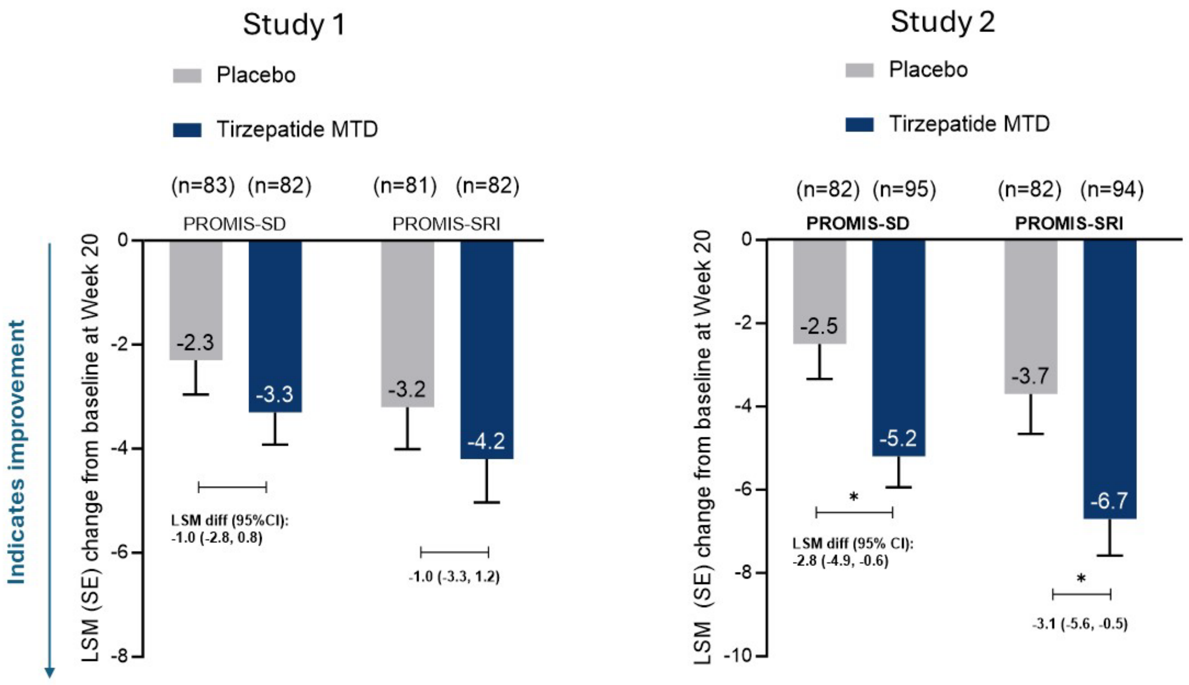

Baseline LSM (SE) scores (Study 1)

	PROMIS-SD	PROMIS-SRI
Placebo	(n=104) 53.8 (0.67)	(n=103) 54.7 (0.82)
TZP MTD	(n=94) 53.9 (0.71)	(n=94) 53.5 (0.86)

Baseline LSM (SE) scores (Study 2)

	PROMIS-SD	PROMIS-SRI
Placebo	(n=98) 56.1 (0.80)	(n=95) 55.2 (0.96)
TZP MTD	(n=105) 56.2 (0.77)	(n=102) 55.6 (0.92)

Abbreviations: CI, confidence interval; diff, difference; LSM, least squares mean; MTD, maximum tolerated dose, n, total number of patients with non-missing data at that particular timepoint; PROMIS-SRI, Patient-Reported Outcomes Measurement Information System Short-Form Sleep-related Impairment 8a; PROMIS-SD, Patient-Reported Outcomes Measurement Information System Short-Form Sleep Disturbance; SE, standard error

Data presented are least-square means derived using analysis of covariance with multiple imputation of missing values. The analysis included the full analysis set for the treatment-regimen estimand.

*p-value <0.05 versus placebo

B. Change in ESS scores

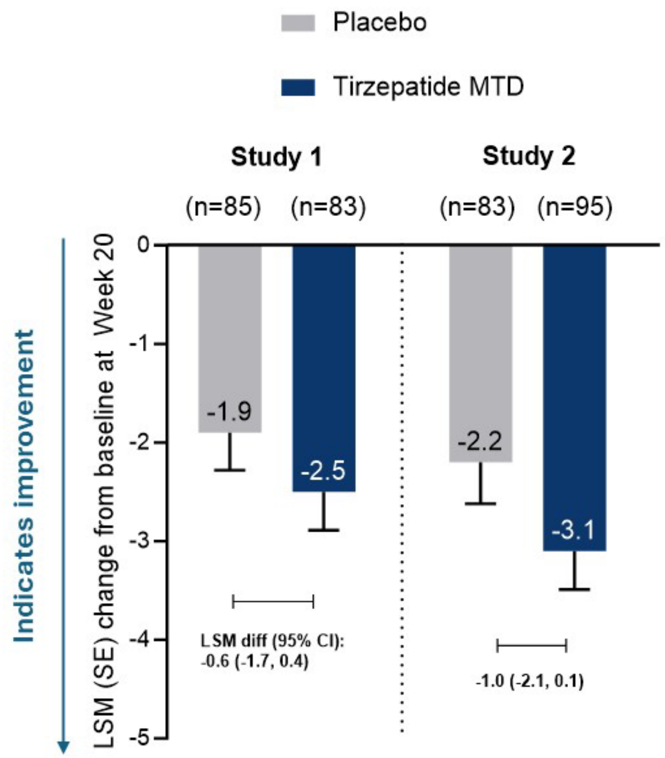

Baseline LSM (SE) scores

	Study 1	Study 2	
Placebo (n=107)	10.7 (0.50)	9.2 (0.45)	Placebo (n=102)
**TZP MTD (n=96)**	**10.3 (0.53)**	**10.8 (0.44)[1]**	**TZP MTD (n=107)**

[1] LSM difference for TZP MTD versus placebo = 1.51 (95% CI: 0.27, 2.76), p=0.018

Abbreviations: CI, confidence interval; diff, difference; ESS, Epworth Sleepiness Scale; LSM, least-square means; MTD, maximum tolerated dose; n, number of participants in the population with baseline and postbaseline value at the specified time point; N, number of participants in the analysis population; SE, standard error; TZP, tirzepatide.

Data presented are least-square means derived using analysis of covariance with multiple imputation of missing values. The analysis included the full analysis set for the treatment-regimen estimand.

*p-value <0.05 versus placebo

C. Change in FOSQ and FOSQ-10 scores

Study 1

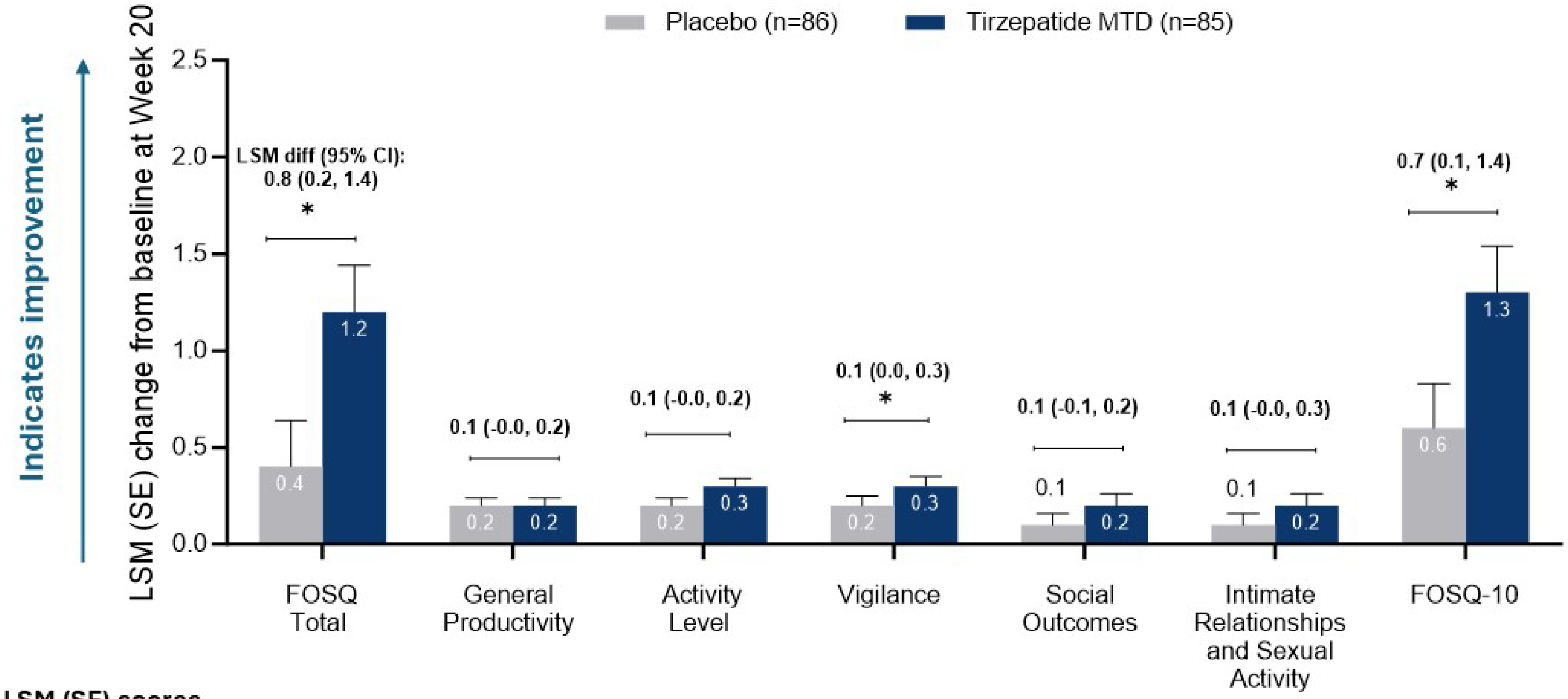

Baseline LSM (SE) scores

	FOSQ Total	General Productivity	Activity Level	Vigilance	Social Outcomes	Intimate Relationships and Sexual Activity	FOSQ-10
Placebo (n=118)	15.8 (0.27)	3.4 (0.05)	3.0 (0.06)	3.1 (0.06)	3.5 (0.07)	3.3 (0.07)	15.2 (0.28)
TZP MTD (n=110)	16.2 (0.28)	3.4 (0.05)	3.1 (0.06)	3.2 (0.07)	3.5 (0.07)	3.4 (0.08)	15.6 (0.29)

N values at baseline for Social outcomes: TZP MTD: n=108; Intimate Relationships and Sexual Activity: Placebo n=102, TZP MTD n=101
N values at Week 20 for Vigilance: Placebo: n=85; TZP-MTD: n=85; Social outcomes: Placebo: n=83, TZP-MTD: n=84; Intimate Relationships and Sexual Activity: Placebo: n=66, TZP MTD: n=77

Study 2

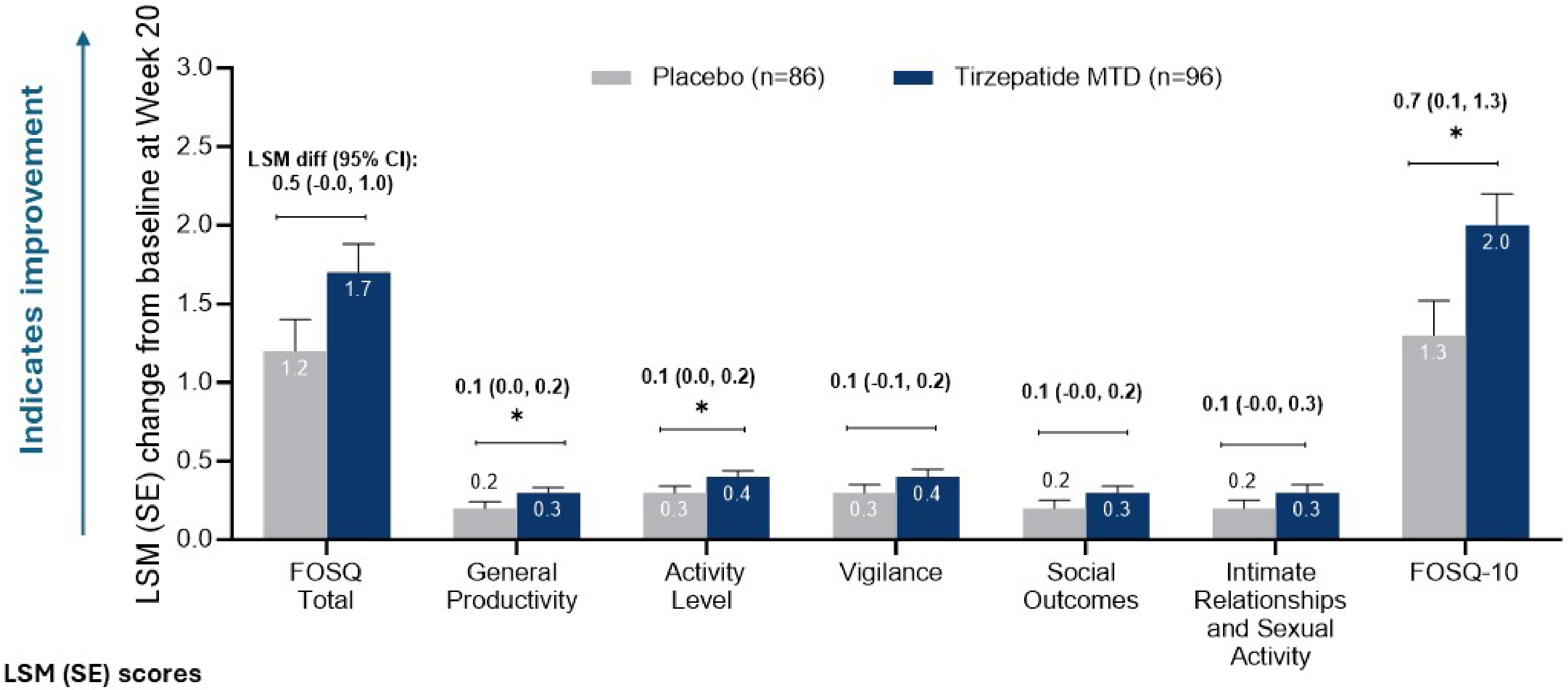

Baseline LSM (SE) scores

	FOSQ Total	General Productivity	Activity Level	Vigilance	Social Outcomes	Intimate Relationships and Sexual Activity	FOSQ-10
Placebo (n=112)	16.5 (0.27)	3.4 (0.05)	3.1 (0.06)	3.2 (0.06)	3.5 (0.07)	3.3 (0.08)	16.1 (0.29)
**TZP MTD (n=117)**	**16.3 (0.27)**	**3.4 (0.05)**	**3.0 (0.06)**	**3.1 (0.06)**	**3.5 (0.07)**	**3.3 (0.08)**	**15.8 (0.28)**

N values at baseline for Social outcomes: Placebo: n=109, TZP MTD: n=111; Intimate Relationships and Sexual Activity: Placebo: n=103, TZP MTD: n=102
N values at Week 20 for Social outcomes: Placebo: n=84, TZP-MTD: n=93; Intimate Relationships and Sexual Activity: Placebo: n=73, TZP MTD: n=84
Abbreviations: CI, confidence interval; diff, difference; FOSQ, Functional Outcomes of Sleep Questionnaire; LSM, least-square means; MTD, maximum tolerated dose; n, number of participants in the population with baseline and postbaseline value at the specified time point; N, number of participants in the analysis population; SE, standard error.
Data presented are least-square means derived using analysis of covariance with multiple imputation of missing values. The analysis included the full analysis set for the treatment-regimen estimand.
General productivity, Activity Level, Vigilance, Social Outcomes, and Intimate Relationships and Sexual Activity are all domains of FOSQ.
*p-value <0.05 versus placebo

D. Change in SF-36v2 scores

Study 1

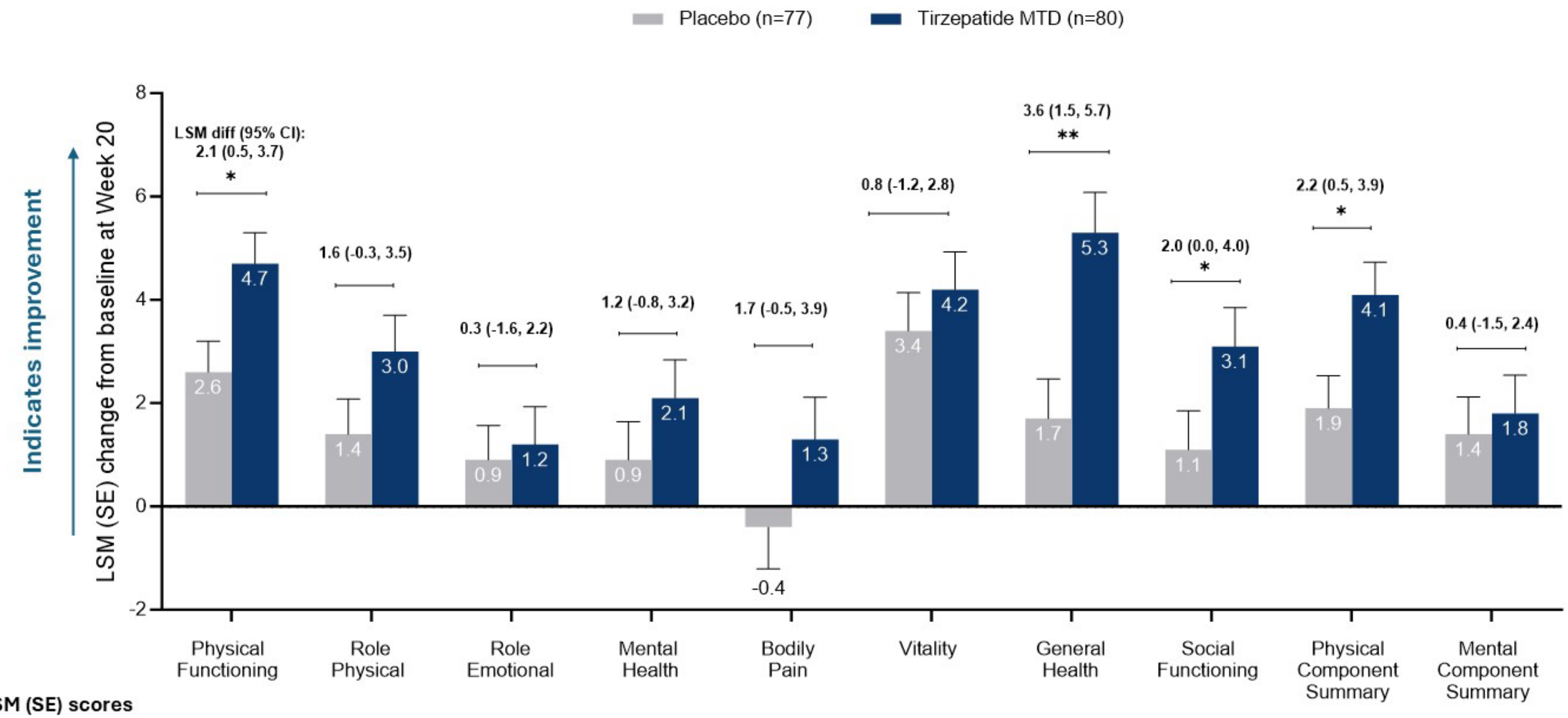

Baseline LSM (SE) scores

	Physical Functioning	Role Physical	Role Emotional	Mental Health	Bodily Pain	Vitality	General Health	Social Functioning	Physical Component Summary	Mental Component Summary
Placebo (n=99)	45.6 (0.83)	47.4 (0.75)	49.1 (0.88)	49.3 (0.86)	48.5 (0.95)	47.2 (0.94)	47.1 (0.88)	47.4 (0.85)	46.3 (0.79)	49.4 (0.92)
**TZP MTD (n=92)**	**47.2 (0.86)**	**49.2 (0.78)**	**49.5 (0.91)**	**49.1 (0.89)**	**50.0 (0.98)**	**48.0 (0.98)**	**46.4 (0.91)**	**49.9 (0.88)[1]**	**47.9 (0.81)**	**49.7 (0.95)**

[1] LSM difference for TZP MTD versus placebo = 2.5 (95% CI: 0.09, 4.94), p=0.042

Study 2

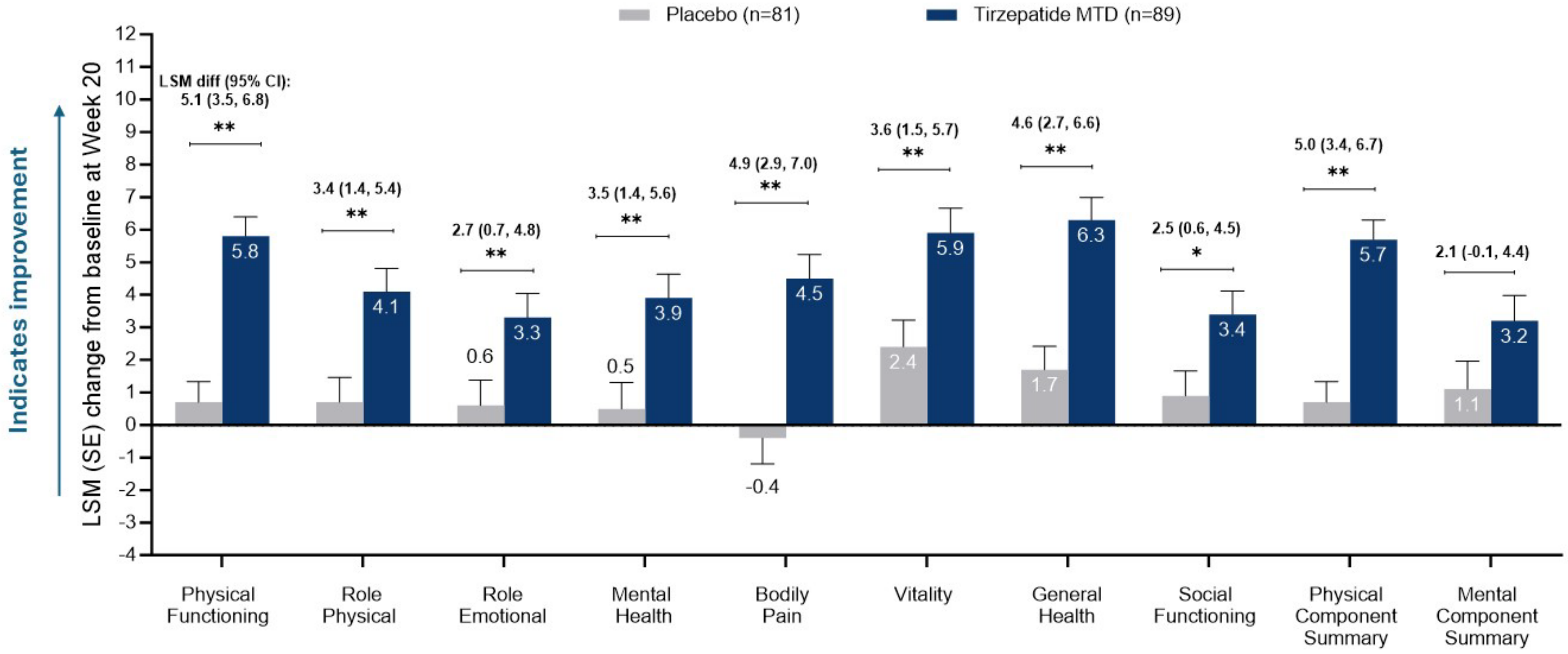

Baseline LSM (SE) scores

	Physical Functioning	Role Physical	Role Emotional	Mental Health	Bodily Pain	Vitality	General Health	Social Functioning	Physical Component Summary	Mental Component Summary
Placebo (n=87)	46.5 (0.93)	46.7 (0.89)	49.4 (0.88)	50.8 (0.83)	50.9 (1.00)	47.8 (1.02)	47.4 (0.99)	49.3 (0.88)	46.9 (0.92)	50.6 (0.90)
TZP MTD (n=87)	48.4 (0.93)	48.4 (0.89)	49.6 (0.88)	49.1 (0.83)	49.5 (1.00)	47.1 (1.02)	46.6 (0.99)	50.1 (0.88)	47.92 (0.92)	49.4 (0.90)

Abbreviations: CI, confidence interval; diff, difference; LSM, least-square means; MTD, maximum tolerated dose; n, number of participants in the population with baseline and postbaseline value at the specified time point; N, number of participants in the analysis population; SF-36 v2, Short-Form-36 Health Survey, Version 2; SE, standard error.

Data presented are least-square means derived using analysis of covariance with multiple imputation of missing values. The analysis included the full analysis set for the treatment-regimen estimand.

*p-value <0.05 versus placebo, **p-value <0.01 versus placebo

E. Shift plots of PGIS scales at Week 20

Study 1

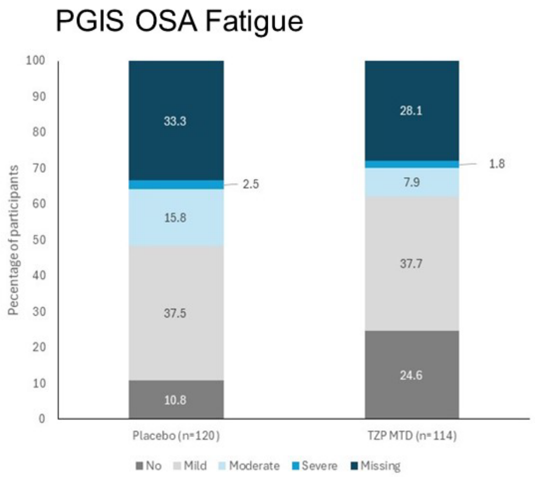

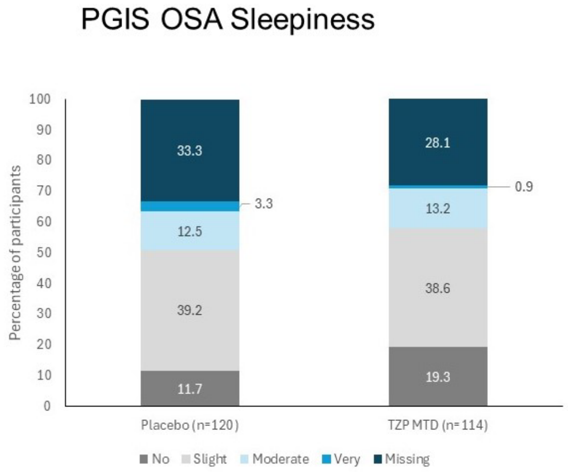

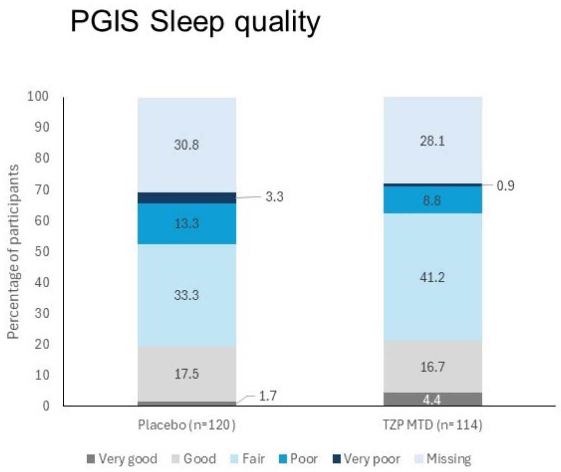

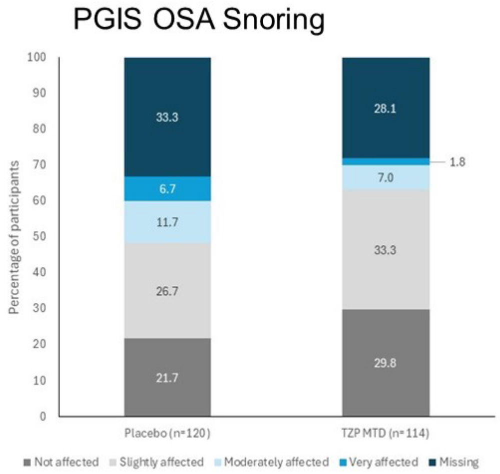

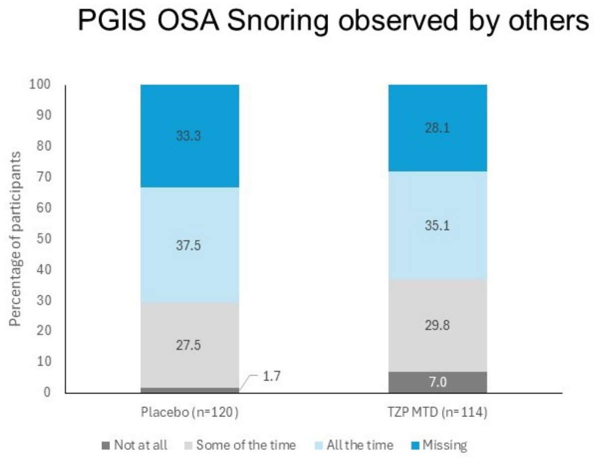

Study 2

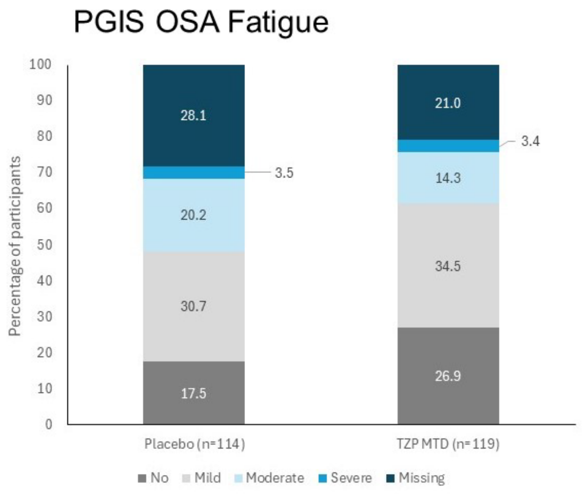

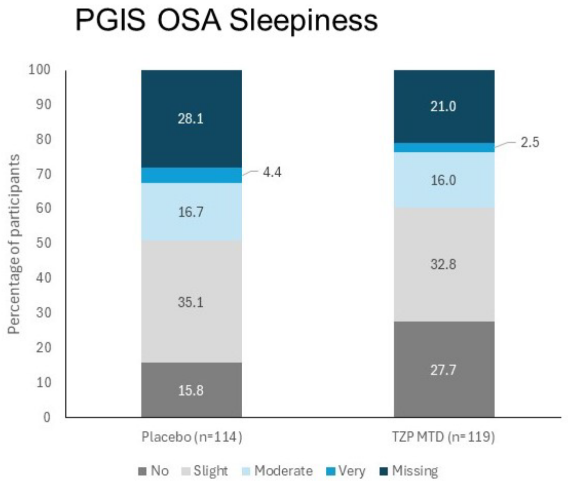

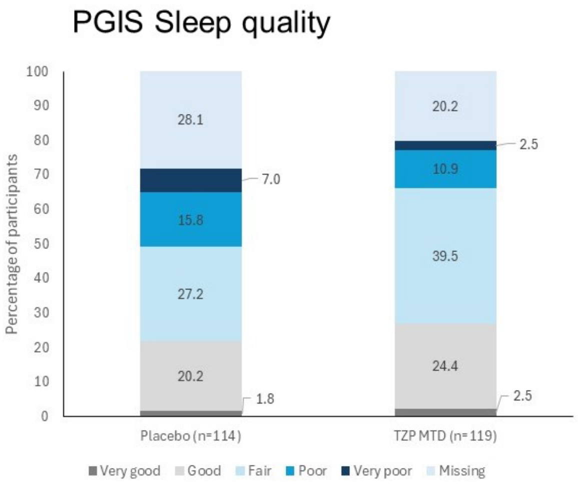

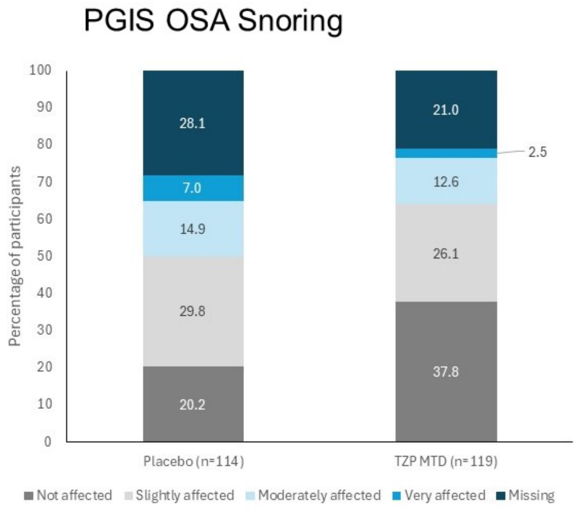

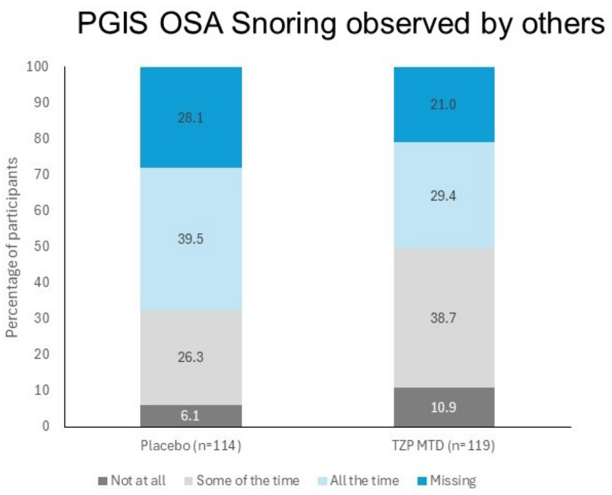

Abbreviations: MTD, maximum tolerated dose; n, number of participants in the population with baseline and postbaseline value; PGIS-OSA, Patient Global Impression of Status – Obstructive Sleep Apnea; TZP, tirzepatide

F. Shift plots of PGIC scales at Week 20

Study 1

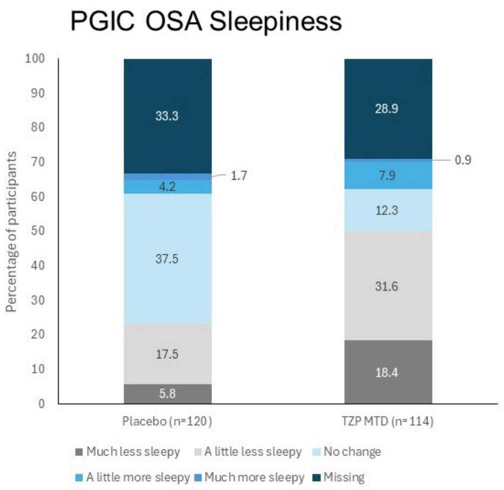

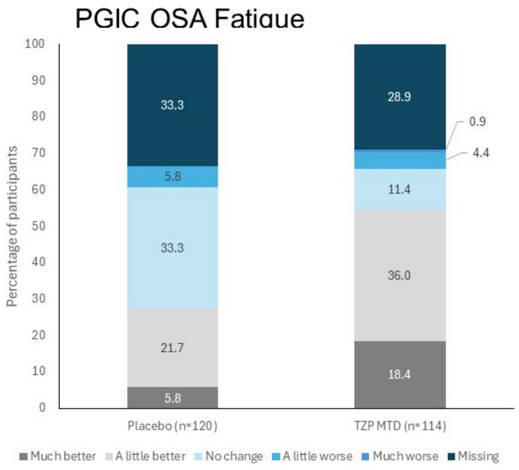

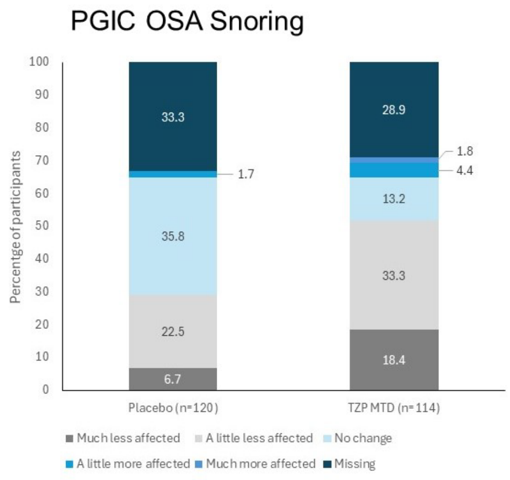

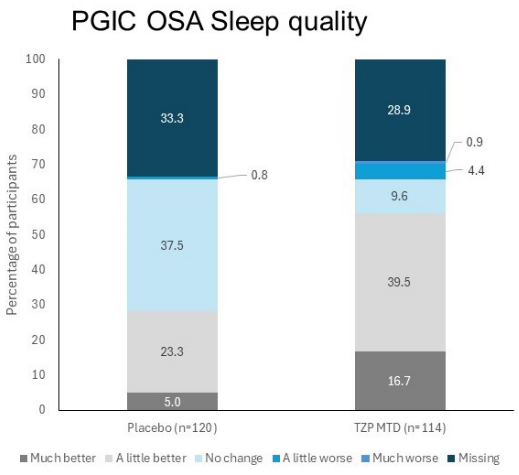

Study 2

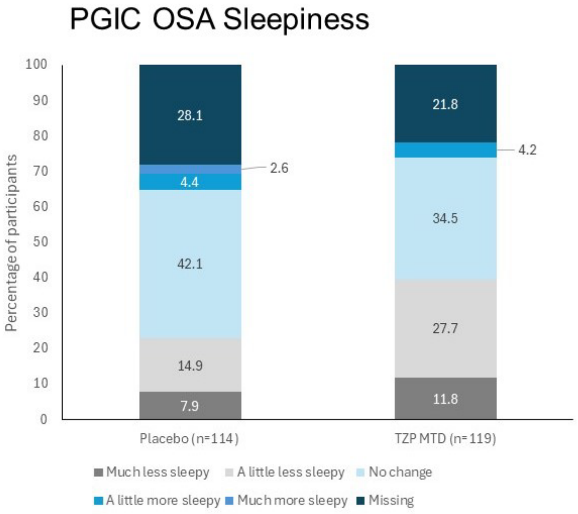
PGIC OSA Sleepiness
Percentage of participants
100
90
80
70
60
50
40
30
20
10
0
28.1
2.6
4.4
42.1
14.9
7.9
21.8
4.2
34.5
27.7
11.8
Placebo (n=114)
TZP MTD (n=119)
Much less sleepy
A little less sleepy
No change
A little more sleepy
Much more sleepy
Missing

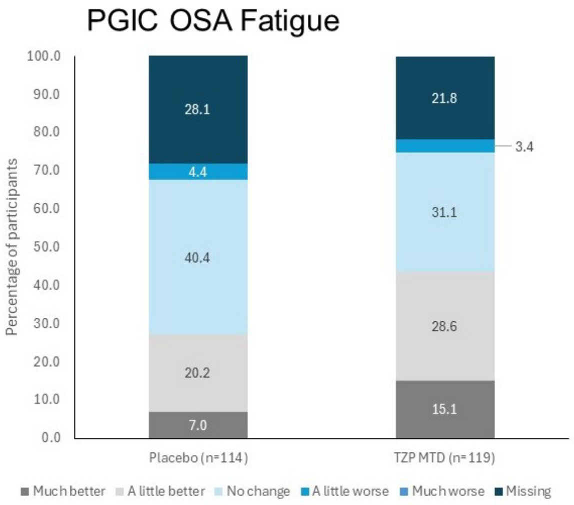
PGIC OSA Fatigue
Percentage of participants
100.0
90.0
80.0
70.0
60.0
50.0
40.0
30.0
20.0
10.0
0.0
28.1
4.4
40.4
20.2
7.0
21.8
3.4
31.1
28.6
15.1
Placebo (n=114)
TZP MTD (n=119)
Much better
A little better
No change
A little worse
Much worse
Missing

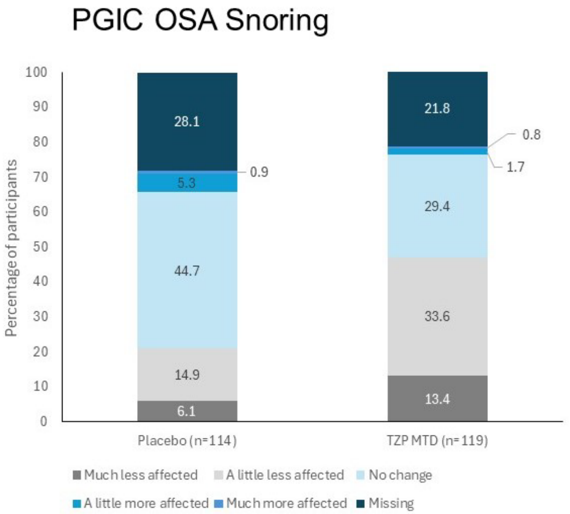
PGIC OSA Snoring
Percentage of participants
100
90
80
70
60
50
40
30
20
10
0
28.1
0.9
5.3
44.7
14.9
6.1
21.8
0.8
1.7
29.4
33.6
13.4
Placebo (n=114)
TZP MTD (n=119)
Much less affected
A little less affected
No change
A little more affected
Much more affected
Missing

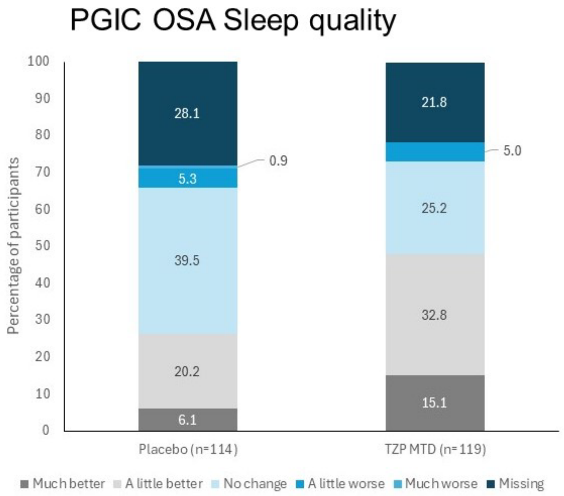
PGIC OSA Sleep quality
Percentage of participants
100
90
80
70
60
50
40
30
20
10
0
28.1
0.9
5.3
39.5
20.2
6.1
21.8
5.0
25.2
32.8
15.1
Placebo (n=114)
TZP MTD (n=119)
Much better
A little better
No change
A little worse
Much worse
Missing

Abbreviations: MTD, maximum tolerated dose; n, number of participants in the population with baseline and postbaseline value; PGIC-OSA, Patient Global Impression of Change – Obstructive Sleep Apnea; TZP, tirzepatide
